# Caveolin-1 is critical for hepatic iron storage capacity in the development of nonalcoholic fatty liver disease

**DOI:** 10.1186/s40779-023-00487-3

**Published:** 2023-11-08

**Authors:** Guang-Hui Deng, Chao-Feng Wu, Yun-Jia Li, Hao Shi, Wei-Chao Zhong, Mu-Keng Hong, Jun-Jie Li, Jia-Min Zhao, Chang Liu, Meng-Chen Qin, Zhi-Yun Zeng, Wei-Min Zhang, Ken Kin Lam Yung, Zhi-Ping Lv, Lei Gao

**Affiliations:** 1 Department of Gastroenterology, Integrated Hospital of Traditional Chinese Medicine, Southern Medical University, Guangzhou, 510315 China; 2 School of Traditional Chinese Medicine, Southern Medical University, Guangzhou, 510515 China; 3 Department of Hepatology, Cancer Center, Integrated Hospital of Traditional Chinese Medicine, Southern Medical University, Guangzhou, 510315 China; 4 Shenzhen Traditional Chinese Medicine Hospital, Shenzhen, 518033 Guangdong China; 5 Department of Biology, Hong Kong Baptist University, Kowloon Tong, Kowloon, Hong Kong SAR, 999077 China

**Keywords:** Caveolin-1, Nonalcoholic fatty liver disease, Iron metabolism, Ferritin, Oxidative stress

## Abstract

**Background:**

Nonalcoholic fatty liver disease (NAFLD) is associated with disordered lipid and iron metabolism. Our previous study has substantiated the pivotal role of Caveolin-1 (Cav-1) in protecting hepatocytes and mediating iron metabolism in the liver. This study aimed to explore the specific mechanisms underlying the regulation of iron metabolism by Cav-1 in NAFLD.

**Methods:**

Hepatocyte-specific *Cav-1* overexpression mice and knockout mice were used in this study. *Cav-1*-knockdown of RAW264.7 cells and mouse primary hepatocytes were performed to verify the changes in vitro. Moreover, a high-fat diet and palmitic acid plus oleic acid treatment were utilized to construct a NAFLD model in vivo and in vitro, respectively, while a high-iron diet was used to construct an in vivo iron overload model. Besides, iron concentration, the expression of Cav-1 and iron metabolism-related proteins in liver tissue or serum were detected using iron assay kit, Prussian blue staining, Western blotting, immunofluorescence staining, immunohistochemical staining and ELISA. The related indicators of lipid metabolism and oxidative stress were evaluated by the corresponding reagent kit and staining.

**Results:**

Significant disorder of lipid and iron metabolism occurred in NAFLD. The expression of Cav-1 was decreased in NAFLD hepatocytes (*P* < 0.05), accompanied by iron metabolism disorder. Cav-1 enhanced the iron storage capacity of hepatocytes by activating the ferritin light chain/ferritin heavy chain pathway in NAFLD, subsequently alleviating the oxidative stress induced by excess ferrous ions in the liver. Further, CD68^+^CD163^+^ macrophages expressing Cav-1 were found to accelerate iron accumulation in the liver, which was contrary to the effect of Cav-1 in hepatocytes. Positive correlations were also observed between the serum Cav-1 concentration and the serum iron-related protein levels in NAFLD patients and healthy volunteers (*P* < 0.05).

**Conclusions:**

These findings confirm that Cav-1 is an essential target protein that regulates iron and lipid metabolic homeostasis. It is a pivotal molecule for predicting and protecting against the development of NAFLD.

**Supplementary Information:**

The online version contains supplementary material available at 10.1186/s40779-023-00487-3.

## Background

Fatty liver disease stands as one of the most prevalent liver diseases globally, affecting a substantial population [1]. Nonalcoholic fatty liver disease (NAFLD), typically initiates with the accumulation of lipids in the liver, followed by the gradual emergence of an evident inflammatory response and liver fibrosis. During this phase, hepatocyte injury or death triggers additional inflammatory responses and eventually aggravates the progression of NAFLD [2, 3]. Therefore, alleviating hepatocyte damage is considered a pivotal strategy to impede the advancement of NAFLD. Epidemiological evidence suggests that the global prevalence of NAFLD reached 25% in 2016 [4]. However, the US Food and Drug Administration has not yet approved any pharmaceutical interventions for NAFLD [5]. Given its important clinical and social value, the mechanism underlying NAFLD development requires further exploration.

Disordered iron metabolism is considered a contributing factor to hepatocyte injury and death in NAFLD. Studies have shown an association between NAFLD development and both iron overload [6] and iron deficiency [7]. These seemingly contrasting findings could be attributed to differences in the severity of NAFLD, the stage of disease progression, and the singularity of the disease. Nonetheless, iron overload is more commonly observed in obese individuals, who are more susceptible to fatty liver disease [8].

Excess ferrous ions (Fe^2+^) generate large amounts of toxic reactive oxygen species (ROS) through the Fenton reaction [9]. These ROS directly cause damage to cellular DNA, lipids, and proteins [10, 11], ultimately leading to cell death via inhibition of adenosine triphosphate, nicotinamide adenine dinucleotide, and glutathione production. Additionally, ferritin is a protein that consists of 24 subunits, including heavy chains (FTHs) and light chains (FTLs) [12]. These subunits construct a cage-shaped complex, which combines and stores ferric irons (Fe^3+^) in an inert form, thus limiting the generation of destructive redox species [13]. The main mechanism underlying iron storage in hepatocytes and macrophages is through the binding of iron to ferritin [14]. The iron chelator deferoxamine mesylate (DFOM) has been reported to attenuate liver injury in NAFLD by decreasing the mitochondrial iron content [15]. Thus, disruptions in iron metabolism play a crucial role in the initiation and progression of NAFLD. So, we speculate that proteins that regulate iron metabolism will play an important role in progression of NAFLD.

Caveolin-1 (Cav-1), a structural and signaling protein on the cytoplasmic membrane, plays vital roles in various liver diseases [16, 17], including a spectrum of conditions such as alcoholic fatty liver disease, NAFLD, fibrosis, liver cirrhosis, and hepatocellular carcinoma [18]. Our previous study confirmed that Cav-1 alleviates ferroptosis in autoimmune hepatitis by suppressing hepatic iron accumulation [19]. However, the relationship between Cav-1 and iron metabolism-related proteins has not been well-elucidated, especially in NAFLD. Therefore, to verify the regulatory mechanism of Cav-1 on liver iron metabolism, we constructed mouse NAFLD model and iron overload model using a high-fat diet (HFD) and high-iron diet, respectively. Meanwhile, RAW264.7 cells and mouse primary hepatocytes were applied to validate our study in vitro experiment.

## Materials and methods

### Animal

A total of 34 C57BL/6J wild-type (WT) mice (Cav-1^+/+^) were obtained from the Guangdong Medical Laboratory Animal Center [SCXK (Guangdong) 2018-0002]. A total of 27 hepatocyte-specific conditional *Cav-1* overexpression mice (AAV9^*Cav-1*^) were generated by adeno-associated virus 9 (AAV9, element sequence of vectors: BGP-MCS-EGFP-3flag-SV40 PolyA; 100 μl/mouse; virus titer: 1 × 10^11^), corresponding a total of 27 negative control mice (AAV9^*NC*^) were generated by negative control AAV9 (100 μl/mouse; virus titer: 1 × 10^11^; Genechem Co., Ltd., Shanghai, China).

Cav-1^*flox/flox*^ mice (Flox mice; purchased from Cyagen Biosciences) were mated with albumin-Cre mice (Alb^*Cre*^; purchased from Cyagen Biosciences) to generate hepatocyte-specific *Cav-1* knockout mice (*Cav-1*-CKO mice) according to the Flox/Cre system (a total of 12 *Cav-1*-CKO mice were utilized in this study); A total of 12 Flox mice were utilized as controls. Primers were designed to identify the genotypes of the mice; loxP primers (Beijing Genomics Institution) were applied to detect the typing of loxP (Jackson Laboratory, US) insertion sites in the genomes of newborn mice. Cav-1^*flox/flox*^ primers (Beijing Genomics Institution) and Alb^*Cre*^ primer (Beijing Genomics Institution) were used to confirm the *Cav-1*-CKO mice. The sequences employed in the construction of the above mice are listed in Additional file 1: Table S1. Identification of mouse genotypes was performed before experiments. The tail of the mice was cut off and digested with Quick Genotyping Assay Kit (D7283M, Beyotime, China). We assembled PCR reactions with ultra-pure water, PCR Primer Mix, Master Mix, and DNA extracts with proportional allocation. Then, we transferred the PCR reactions to a thermal cycler and ran thermocycling conditions. Finally, we analyzed PCR products by agarose gel electrophoresis.

C57BL/6J WT mice (Cav-1^+/+^), between 8 and 12 weeks of age, were randomly divided into normal diet (NC) group, HFD group, and HFD + DFOM group, consisted with 6 mice in each group. HFD for 12 weeks were utilized to construct NAFLD mice model (Hayek Western-type Diet, No. TP26305, Trophic Animal Feed High-tech Co., Ltd., China; HFD caloric content: fat 42%, protein 14%, carbohydrate 44%; cholesterol content 0.2%). NC group mice were fed with low-fat diet (No. TP26362, Trophic Animal Feed High-tech Co., Ltd., China; caloric content: fat 10%, protein 20%, carbohydrate 70%). In parallel studies, mice were treated with iron chelator DFOM (100 mg/kg, S5742, Selleck, China) or an equal volume of normal saline intraperitoneally 3 times per week from the 7th week.

High-iron diet containing carbonyl iron for 12 weeks (AIN93 standard, 2.5% carbonyl iron, No. TP0457G, Trophic Animal Feed High-tech Co., Ltd., China) was used to construct the iron overload mice model. The control group mice were provided with the standard control diet for feeding (No. LAD3001G, Trophic Animal Feed High-tech Co., Ltd., China). Approval for the animal experiment program was granted by the Experimental Animal Ethics Committee at Southern Medical University (No. L2020044).

The mice were kept in a pathogen-free facility at the Southern Medical University under consistent environmental conditions, including a humidity level of (50 ± 5)%, a temperature of (24 ± 1) °C, a 12-h light–dark cycle, and unrestricted access to food and water.

### Culture of primary hepatocyte and RAW264.7 cell and construction of NAFLD model

Primary hepatocytes were extracted from the liver C57BL/6 mice according to a published protocol [20] with slight modification. In brief, the mice were anesthetized with isoflurane inhalation, followed by a surgical incision of the abdominal cavity to expose the portal vein. HBSS-EGTA buffer and type IV collagenase digestion solution were prepared in advance and rewarmed in a 37 ℃ before use. The preheated HBSS-EGTA buffer (30 ml/mouse) was poured into the liver through the portal vein. Then assistant replaced the preheated type IV collagenase digestive solution (50 ml/mice). The liver tissue was removed and gently ground, and liver cell suspension was transferred to a 50 ml centrifuge tube through a 70-mesh filter, following by centrifuged at 50 *g* × 1 min and washed 3 times with DMEM medium [supplemented with 1% penicillin, 1% streptomycin (Gibco, Carlsbad, CA, USA), and 10% fetal bovine serum (FBS)]. For each independent experiment, Trypan blue was utilized to assess cell viability, which was consistently maintained at 80%. Hepatocytes (6 × 10^4^ cells/ml) were seeded in 24-well cell culture plate coated with type I collagen (No. C7661-5MG, Sigma-Aldrich, USA) and cultured in the DMEM medium. At 24 h after seeding, hepatocytes were treated with 100 μmol/L palmitic acid (PA; No. P5585-10G, Sigma-Aldrich, USA) and 200 μmol/L oleic acid (OA; No. O1008-1G, Sigma-Aldrich, USA) to construct in vitro NAFLD model [21].

The RAW264.7 cell line was purchased from the National Collection of Authenticated Cell Cultures in China. These cells were cultured in DMEM supplemented with 10% FBS, as well as 1% penicillin–streptomycin. At 24 h after seeding, RAW264.7 cells were treated with 100 μmol/L PA and 200 μmol/L OA to construct NAFLD model [21].

### Human subjects

A total of 52 NAFLD patients recruited from the Shenzhen Traditional Chinese Medicine Hospital, were diagnosed by ultrasonography. We screened suitable clinical trial participants according to the following inclusion criteria and exclusion criteria. Inclusion criteria: (1) male or female, aged from 20 to 60; (2) normal group: no alcohol dependence medical history, equivalent amount of ethanol < 140 g/week in nearly 12 weeks, no single history of heavy drinking (equivalent amount of ethanol > 80 g/d), no drinking in nearly 2 weeks, no significant liver dysfunction or severe diseases; (3) case group: meeting the diagnostic criteria of the research program; and (4) subjects must sign informed consent. Exclusion criteria: (1) subjects have participated in other clinical studies or were infected before this study within 4 weeks; (2) subjects take part in other studies during this study; (3) gravida or lactating female; (4) subjects with history of hypoglycemic syncope and blood phobia; (5) subjects with severe complications of liver diseases, such as spontaneous bacterial peritonitis, hepatic encephalopathy, hepatorenal syndrome, hepatomyocardosis, hepatopulmonary syndrome, hepatic failure and the like; (6) subjects with other severe systemic diseases, such as acquired immune deficiency syndrome, cephalomeningitis, myocardial infarct, renal failure, hemorrhage of digestive tract and the like; and (7) subjects with neurological or psychiatric diseases, such as paralysis agitans and major depressive disorder; researchers exclude subjects from this study because of other reasons.

A total of 29 healthy volunteers’ serum was obtained from volunteers without NAFLD. Volunteers with one of these indicators were excluded from the study: known acute or chronic disease, excessive alcohol consumption (> 140 g for men or > 70 g for women, per week), drug or toxin use, or viral infection [22]. Finally, 6 patients with NAFLD were excluded, and 5 healthy volunteers were excluded. The human study was approved by the Ethics Committee of Shenzhen Traditional Chinese Medicine Hospital (Clinical No. SZTCMH-E-2014-4), Traditional Chinese Medicine University of Guangzhou, and adhered to the principles of the Declaration of Helsinki (Registration No. ChiCTR-ROC-15007195). Written informed consent was obtained from all volunteers or their families.

### Construction of *Cav-1* gene modified cells by lentivirus

Cell suspensions with a density of (3 − 5) × 10^5^ cells/ml were prepared using complete medium (10% FBS + DMEM + 1% penicillin–streptomycin antibiotic), and 500 μl cell suspensions were inoculated into 24-well plates. Twenty-four hours later, the complete medium was replaced, and 20 μl of HitransG P infection enhancement solution was added to each well. The corresponding volume of virus was then added according to the following formula: virus volume = (MOI × number of cells)/virus titer, adding corresponding volume of virus. Sequence for gene identification refers to Additional file 1: Table S2. The culture medium was replaced with complete medium after 8 h. Finally, the optimal concentration of puromycin with the optimal concentration (2 μg/ml) was used for screening.

### Preparation of PA

The method of PA preparation was based on a previous study [20]. The free fatty acids/bovine serum albumin (FFA/BSA) complex was prepared as described previously, with slight modification. Briefly, 100 mmol/L of palmitate or other FFA was prepared in 0.1 mol/L NaOH at 70 °C. In an adjacent water bath at 55 °C, a 10% (wt/vol) FFA-free BSA solution was prepared in DMEM medium. The FFA solution was added dropwise to the BSA solution at 55 °C, and the FFA/BSA mixture was vigorously vortexed for 10 s before it was incubated for a further 10 min at 55 °C. Then, the FFA/BSA complex solution was cooled to room temperature (RT) and sterilized by filtration with a 0.45 μm pore-size membrane filter. The prepared FFA/BSA complex was stored at − 20 °C. For the control groups, a corresponding volume of FFA-free BSA solution was used.

### Detection of the serological indicators

#### Serum alanine aminotransferase (ALT) and aspartate transaminase (AST)

The samples were prepared according to the instructions of the ALT Assay Kit (C009-2-1, Nanjing Jiancheng, China) and AST Assay Kit (C010-2-1, Nanjing Jiancheng, China). The protein concentrations of the samples were determined using a BCA Kit (P0012, Beyotime, China). The samples and stop solution were added to 96-well plates and slowly shaken before incubation for 15 min at RT. Then, the OD value (wavelength = 510 nm) was detected with a microplate tester.

#### Serum total cholesterol (TC)/triglyceride (TG)/low-density lipoprotein-cholesterol (LDL-C)/fasting blood-glucose (FBG)/γ-glutamine (γ-GGT)/total bilirubin (TBiL)/albumin (ALB)/creatinine (Cr)/uric acid (UA)/total iron

Preparing a proper serum sample first. Then the detection reagent was prepared: R1 and R2 were prepared and mixed in accordance with the instruction ratio by single reagent method (TBiL, R1 + R2 = 180 μl + 90 μl; total iron, R1 + R2 = 250 μl + 25 μl); R1 and R2 were respectively applied by double reagent method (TC: R1 = 200 μl, R2 = 100 μl; TG: R1 = 240 μl, R2 = 60 μl; LDL-C: R1 = 300 μl, R2 = 100 μl; FBG: R1 = 300 μl, R2 = 150 μl; γ-GGT: R1 = 200 μl, R2 = 50 μl; ALB: R1 = 300 μl; Cr: R1 = 210 μl, R2 = 70 μl; UA: R1 = 240 μl, R2 = 60 μl). Finally, the corresponding parameters were set on the automatic biochemical analyzer (wavelength: TBiL = 450 nm, ALB = 635 nm, total iron = 578 nm; primary and secondary wavelength: TC = 505/670 nm, TG = 505/670 nm, LDL-C = 570/700 nm, FBG = 505/670 nm, γ-GGT = 405/505 nm, Cr = 540/670 nm, UA = 505/670 nm). According to the procedure provided by the reagent supplier, the samples were loaded in sequence, and the results were determined on the automatic biochemical instrument.

### Iron detection in liver tissue

The iron concentration of liver tissue was detected by an Iron Assay Kit (No. ab83366, Abcam, UK), according to the instructions provided by the manufacturer. Briefly, a standard dilution was prepared in the standard wells (100 μl), and the sample wells (40 μl) were prepared with sample solution (the volume was adjusted to 100 μl/well using iron assay buffer). Then, 5 μl of iron reducer was added to each standard well. The concentration of Fe^2+^ in the liver tissue samples was determined by adding 5 μl of assay buffer to each sample. To determine the total iron (Fe^2+^ + Fe^3+^) concentration of the liver tissue samples, 5 μl of iron reducer was added to each sample. Then, the reagents were mixed and incubated for 30 min in an oven at 37 ℃. Next, 100 μl of iron probe was added into each well and the solution was mixed well and incubated for 60 min in an oven at 37 °C in the dark. Finally, the OD value was detected with a microplate meter (wavelength = 593 nm).

### Slice preparation for histological analysis and lipid drops dyeing

Paraffin sections were prepared as described previously [23]. Liver tissues from mice were perfused with PBS and fixed with 4% paraformaldehyde. The following day, the liver tissues were penetrated with an ethanol gradient and embedded in paraffin. Finally, the paraffin blocks were cut into 4-μm thick sections. The liver tissue sections were stained with HE and Sirius Red stain, according to the manufacturers’ instructions. Pathological changes were observed with an orthographic microscope.

Frozen sections were prepared in accordance with previously described methods [23]. Briefly, the liver tissues were fixed with 4% paraformaldehyde and then dehydrated with 15% and 30% sucrose solutions for 48–72 h, respectively. The prepared liver tissues were embedded in optimal cutting temperature compound (OCT) gel. Finally, the tissues were sliced into 14-μm thick sections to obtain frozen sections.

Oil Red O and Nile Red staining were performed to analyze live lipid accumulation. (1) Oil Red O staining: the frozen sections or cell crawling pieces were washed twice with PBS (5 min/time) and soaked in 100% propylene glycol for 5 min. Subsequently, the frozen sections were incubated in Oil Red solution for 10 min. The sections were then soaked in 85% propylene glycol for 3 min and then washed with PBS twice (5 min/time). After mounting the slides with anti-fluorescence quencher, an upright microscope was used to observe the slides and take pictures. (2) Nile Red staining: the frozen sections were washed twice with PBS (5 min/time). The nuclei were stained with DAPI (Solarbio Life Science, China) for 5 min, and then the sections were washed twice with PBS (5 min/time). Next, the frozen sections were incubated in Nile Red working solution for 10 min. Finally, the sections were washed twice with PBS and mounted with anti-fluorescence quencher. This entire procedure was performed in the dark.

### Immunofluorescent staining

For liver tissue immunofluorescence, the frozen sections were blocked with blocking buffer consisting of PBS, 0.2% Triton X-100 and 5% goat serum solution for 1–2 h. Then, the liver tissues were separately incubated with primary antibodies: Cav-1 (mouse, 1:400, ab17052, Abcam, UK), CD68 (rat, 1:200, ab283316, Abcam, UK), CD163 (rabbit, 1:200, 16646-1-AP, Proteintech, China), Ter-119 (mouse, 1:400, #14-5921-82, ThermoFisher, USA), alpha-smooth muscle actin (α-SMA; rabbit, 1:500, 14395-1-AP, Proteintech, China). After primary antibody incubation, the liver tissues were incubated with secondary antibody, goat anti-rabbit 594-conjugated IgG (1:800, #8889, CST, USA) or goat anti-mouse Alexa Fluor 488-conjugated IgG (1:800, #4408, CST, USA). DAPI was used to stain the nuclei. Finally, the tablets were sealed with anti-fluorescence quenching agent (Dako, Denmark).

For cell immunofluorescence, the cells were rinsed three times with PBS buffer and fixed with 4% paraformaldehyde. Between each of the steps described below, the cells were rinsed with PBS buffer for 5 min three times. The cells were permeabilized with a solution of PBS containing 0.1% Triton X-100 for 10 min and then incubated with blocking solution (PBS containing 5% normal goat serum solution) for 2 h at RT. Then, they were incubated overnight with the primary antibodies appropriately diluted in blocking solution. The incubation was performed in a humidified chamber at 4 °C, and the primary antibodies included Cav-1 (rabbit, 1:400, D46G3, CST, USA). Next, the cells were incubated with the secondary antibody for 2 h. The cells were then counterstained with DAPI for 5 min at RT after being rinsed with PBS buffer for 5 min. Following rinsing with PBS buffer for 5 min, twice, the cell slides were cover-slipped with fluorescent mounting medium (Solarbio, China).

Multiplex fluorescence immunohistochemical staining was performed according to the procedure provided in the kit (abs50012, absin, China). In brief, the paraffin slides were baked in an oven at 65 °C for 30 min, dewaxed with xylene, and then hydrated with alcohol. 10% neutral formalin soak paraffin slides 10 min, double distilled water wash slides1min, repeat 3 times. The paraffin slides were put into the working solution 1 × containing the antigen repair solution, and boiled in the microwave oven at high heat and maintained at low heat for 15 min. Remove the remaining lotion from the slide and block it with blocking buffer. After blocking, the corresponding primary antibody solution was added and incubated at room temperature for 1 h. Then soak the slide with 1 × TBST buffer for 3 min, repeat 2 times. The HRP secondary antibody working solution was added, incubated at room temperature for 10 min, and then soaked the slides with 1 × TBST buffer for 3 min, repeat 2 times. Add 1 × dye working solution 100 μl to the slide and incubate at room temperature for 10 min. 1 × TBST buffer dip slide 3 min, repeat 3 times, microwave repair, 1 × TBST buffer dip paraffin slides 2 min. The first primary antibody stain has been completed here. Subsequent other primary antibodies staining was performed starting from the blocking buffer. After all primary antibodies were stained, 1 × DAPI working solution was added to the sample and incubated at room temperature for 5 min. Soak slides with 1 × TBST for 2 min, repeat 3 times. Finally, the anti-fluorescence quenched sealing slides were added and photographed under confocal fluorescence microscope. Primary antibodies included: Cav-1 (mouse, 1:400, ab17052, Abcam, UK), F4/80 (rabbit, 1:400, #30325, CST, USA), CD68 (rat, 1:200, ab283316, Abcam, UK), heme oxygenase-1 (HO-1; rabbit, 1:400, ab13243, Abcam, UK), ferroportin 1 (Fpn1; rabbit, 1:300, NBP1-21502, NOVUS, USA).

### Immunohistochemical staining

According to previously described methods, the liver tissues were sequentially sliced, dewaxed, and hydrated. Then, sodium citrate was used for antigenic repair. Subsequently, the endogenous peroxidase was removed from the tissues, and they were sealed with 5% goat serum for 1–2 h. Next, the liver tissues were incubated with primary antibodies for 12 h, including CD11b (rabbit, ab133357, Abcam, UK), F4/80 (rabbit, #30325, CST, USA), Cav-1 (mouse, ab17052, Abcam, UK), sterol regulatory element binding transcription factor 1 (SREBP1; rabbit, ab28481, Abcam, UK), FTL (rabbit, 10727-1-AP, Proteintech, China), FTH (rabbit, A19544, ABclonal, China), and 4-hydroxynonenal (4-HNE; rabbit, ab48506, Abcam, UK). The liver tissues were then washed and incubated with goat anti-rabbit/mouse IgG. Chromogen reactions were performed with diaminobenzidine (DAB, Sigma USA). Then, hematoxylin staining was performed. After dehydration with ethanol, the liver tissues were cover-slipped with Canada balsam (Sigma, USA).

### Prussian blue staining

High-iron model liver tissues were stained with a Prussian Blue Iron Stain Kit (No. BB-44371, BestBio, China). Because the liver iron content was significantly increased in the high-iron diet model, a conventional Prussian Blue Staining Kit was used to stain the tissues. The paraffin-embedded sections were de-waxed in water and then washed with distilled water for 1 min. After staining with reagent A for 20 min, the sections were rinsed thoroughly with distilled water for 5 min. The cell nuclei were lightly stained with nuclear red reagent B for 6 min and then washed with water. Finally, routine dehydration and clearing were performed, followed by sealing with neutral mounting medium. Positive staining appeared as a blue color.

NAFLD model liver tissues were stained with a Prussian Blue Iron Stain Kit (Enhanced with DAB, No. G1428, Solarbio, China). Due to the lower iron content of the liver in the NAFLD model compared to the high-iron model, an enhanced version of the Prussian Blue Staining Kit was used for staining. The kit was rewarmed for 20 min to RT (25–30 ℃). The Perls working solution was prepared at a ratio of 1:1, and was then dropped onto the slices. The slides were incubated in a wet box at 37 ℃ for 20 min. Then, they were rinsed with distilled water and the incubation working solution was dropped onto them, at a ratio of 1:9. The slices were then incubated at 37 ℃ for 10–20 min. Next, the slices were gently soaked in 1 × PBS and enhanced working solution was dropped onto them at a ratio of 1:1:18. The slices were then incubated at 37 ℃ for 10–20 min. Subsequently, the slices were stained with redyeing solution for 3–5 min and soaked in distilled water for 10 min. After staining, the slices were dehydrated in gradient ethanol, cleared with xylene, and sealed with resinene. A positive result was indicated by yellowish-brown staining.

### Western blotting analysis

RIPA cell lysis buffer (Sigma, USA) containing a protease inhibitor cocktail (Sigma, USA) and phosphatase inhibitor cocktail (Sigma, USA) was used to extract proteins. When we had finished quantification and denaturation, 20–40 μg protein samples were loaded onto sodium dodecyl sulfate–polyacrylamide gels and transferred to polyvinylidene fluoride membranes (PVDF; Millipore, USA). With BSA blocking, the PVDF were incubated overnight at 4 °C with primary antibodies and then with horseradish peroxidase-conjugated secondary antibodies. ECL detection reagents were used to detect the expression of related proteins (Millipore, USA), for example: Cav-1 (rabbit, #3238, CST, USA), GAPDH (rabbit, #8884, Abcam, UK), β-actin (mouse, 60008-1-Ig, Proteintech, China), HO-1 (rabbit, ab13243, Abcam, UK), nuclear factor erythroid 2-related factor 2 (Nrf2; rabbit, 16396-1-AP, Proteintech, China), Fpn1 (rabbit, NBP1-21502, NOVUS, USA), FTL (rabbit, 10727-1-AP, Proteintech, China), FTH (rabbit, A19544, ABclonal, China), and fatty acid synthase (FASN; rabbit, ab128870, Abcam).

### ELISA detection

The ELISA kits [Cav-1 ELISA Kit, No. BS-E19429M1, JSBOSSEN, China; hepcidin ELISA Kit, No. BS-E9157M1, JSBOSSEN, China; ferritin ELISA Kit, No. BS-E9156M1, JSBOSSEN, China; transferrin (Tf) ELISA Kit, No. BS-E5398H1, JSBOSSEN, China] were placed at RT for 20 min in advance. Then, 20 × washing buffer was diluted 20 times with distilled water to obtain 1 × washing buffer. Standard and sample wells were set according to the actual quantity, and 50 μl of the corresponding concentration of standard solution was added into the standard wells. Subsequently, 50 μl of serum was seeded into the sample wells. The blank well was left alone. Then, 100 μl of horseradish peroxidase-labeled detection antibody was added to the standard and sample wells, and the reaction well was sealed with a sealing plate membrane. The plate was then heated in a water bath at 37 ℃ for 60 min. Subsequently, the liquid was removed from the reaction wells. Finally, 50 μl of substrate A and substrate B were added to each well and the plate was incubated at 37 ℃ for 15 min in the dark. After incubation, 50 μl of stop solution was added to each well, and the OD value was examined within 15 min at a wavelength of 450 nm using a microplate meter.

### Detection of superoxide dismutase (SOD) in liver tissue

An appropriate amount of liver tissue was obtained and added to pre-cooled PBS for homogenization (1 mg of tissue was added to 10 μl PBS). Then, the homogenate was centrifuged at 4 ℃ and the supernatant was obtained for later use. A BCA Kit was used to determine the protein concentration of the supernatant. Next, the sample was diluted 20 times with SOD detection buffer, according to the protein concentration. An appropriate amount of WST-8/enzyme working solution (151 μl SOD detection buffer + 8 μl WST-8 + 1 μl enzyme solution) was prepared to a volume of 160 μl for each reaction. The reaction starter solution was prepared by adding 39 μl of SOD detection buffer for every 1 μl of reaction starter solution (40×). After adding the sample and various other solutions successively to a 96-well plate, the working solution was added to start the reaction and the plate was incubated in an oven at 37 ℃ for 30 min. Finally, the absorbance was measured with an enzyme reader at 450 nm and 600 nm.

### Transmission electron microscopy detection

The liver tissue was removed immediately after anesthesia, and then was placed in pre-cooled 2.5% glutaraldehyde. The left liver was quickly cut into 1 mm^3^ pieces and placed in 2.5% glutaraldehyde for incubation at RT for 1 h, and then transferred and incubated overnight in a refrigerator at 4 ℃. The next day, liver tissue was washed with PBS and fixed with 1% percolating acid at 4 ℃ for 3 h, then dehydrated in ethanol and acetone successively, and embedded in epoxy resin for 4 h. Finally, the prepared tissue was cut into 70 nm sections, stained with uranium dioxane acetate and lead citrate, and observed and photographed by Hitachi H-7500 transmission electron microscope.

### Flow cytometry analysis

Hepatic non-parenchymal cells (HNPC) were isolated according to our previously described method [24]. Briefly, type IV collagenase was used to perfuse the liver at a concentration of 25 mg/mouse. Then, the separation of other hepatocytes and non-hepatocytes (HNPC) was performed based on density gradients, and the macrophages were extracted. Next, the concentration of the cell suspensions was diluted to 1 × 10^6^ cells/100 μl in fluorescence-activated cell sorting (FACS) tubes. The following monoclonal antibodies were used to mark the macrophages: PE-conjugated anti-F4/80 (BD Biosciences, San Diego, CA, USA) and FITC-conjugated anti-CD11b (BD Biosciences, San Diego, CA, USA). Nonspecific Fc-mediated interactions were blocked by incubation with CD16/32 (BD Biosciences, San Diego, CA, USA). 7-aminoactinomycin D (BD Biosciences, San Diego, CA, USA) staining was used to separate the dead cells. Voltages were set to accommodate unstained cells, and compensation was set based on single-stained positive controls for each color. FACS (BD LSRFortessa X-20, CA, USA) was utilized to sort the cells, and FlowJo software (Tree Star, Ashland, OR, USA) was used to analyze the results.

### Detection of CCK-8 assay

Primary hepatocytes were collected and 100 μl was add into each hole of the 96-well plate to make the cell density to be measured 3 × 10^3^–5 × 10^3^ cells/well. Then, cells were cultured at 37 ℃ for 24 h with 5% CO_2_, and added 10 μl CCK-8 solution to each well. We continued to incubate in the cell incubator for 30 min to 4 h. Finally, the absorbance of each hole was measured by enzyme marker at 450 nm every 30 min.

### Statistical analysis

Animal experiments were performed by at least three independent individuals in each group, whereas cell experiments were performed by at least three independent repeated experiments. The data are presented as the means ± standard deviation (SD). Statistical differences were evaluated by an *t* test or one-way analysis of variance, followed by Tukey’s multiple comparisons test on dependent experimental designs. Correlation tests between continuous variables were performed by Pearson’s coefficient for linear regression. Spearman’s rank correlation test was used if the regression was nonlinear. To assess the diagnostic performance of serum biomarkers, receiver operating characteristic curve analysis was performed, and the area under the receiver operating characteristic curve (AUROC) was utilized to evaluate the predictive power. The AUROCs were compared using the Wilson/Brown. All analyses were performed using GraphPad Prism software (San Diego, CA). Values of *P* < 0.05 were considered statistically significant.

## Results

### Cav-1 was down-regulated and was accompanied by abnormal lipid and iron metabolism in the NAFLD model

**Fig. 1:**
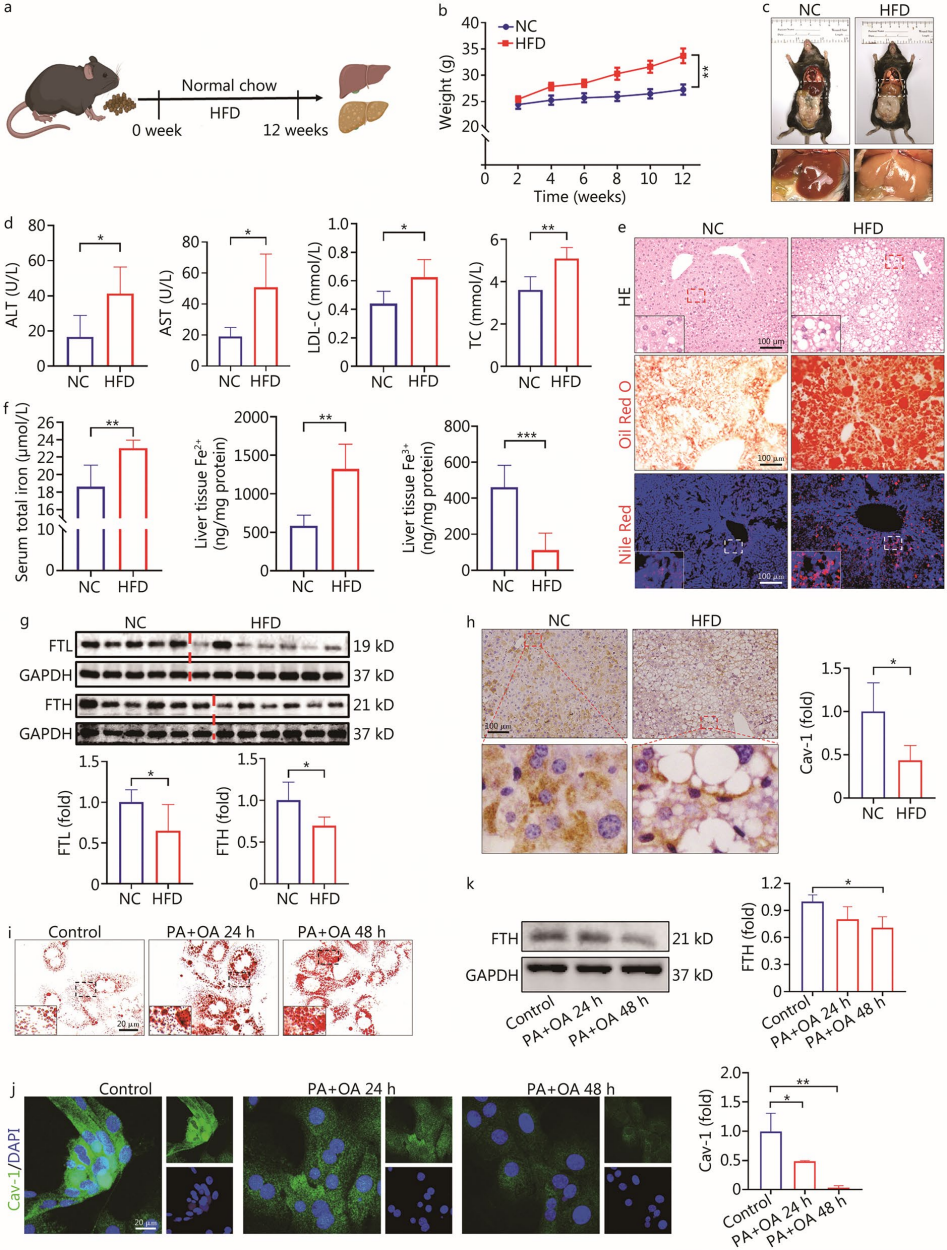
NAFLD was characterized by abnormal iron metabolism accompanied by down-regulation of Cav-1 in liver. **a** Flow chart of mouse NAFLD model construction. **b** Line chart of changes in body weight of mice within 12 weeks (*n* = 7). **c** Accumulation of fat in liver of mice. **d** Serum levels of ALT, AST, LDL-C and TC between NC group and HFD group *(n* = 5). **e** HE staining, Oil Red O staining and Nile Red staining results of liver tissue between NC group and HFD group. **f** Concentration of serum total iron, liver tissue Fe^2+^ and liver tissue Fe^3+^ between NC group and HFD group (*n* = 5). **g** Western blotting analyses of FTL and FTH proteins between NC group and HFD group (*n* ≥ 5). **h** Cav-1 immunohistochemistry staining between NC group and HFD group (*n* = 4). **i** Oil Red O staining results of primary hepatocytes with or without 100 μmol/L PA + 200 μmol/L OA. **j** Cav-1 immunofluorescence staining of primary hepatocytes with or without PA + OA (*n* = 3). **k** Western blotting analyses of FTH proteins with or without PA + OA (*n* = 3). ^*^P < 0.05, ^**^P < 0.01, ^***^P < 0.001, as determined by Student’s *t* test analysis or one-way ANOVA with Bonferroni post hoc analysis. All data were shown as the mean ± SD. NAFLD non-alcoholic fatty liver disease, NC negative control, HFD high-fat diet, ALT alanine aminotransferase, AST aspartate transaminase, LDL-C low-density lipoprotein cholesterol, TC total cholesterol, FTL ferritin light chain, FTH ferritin heavy chain, Cav-1 caveolin-1, PA palmitic acid, OA oleic acid

The process of construction of the mouse NAFLD model is shown in Fig. 1a. Over time, compared with the NC group, the body weight of NAFLD mice was significantly increased from the 4th week (*P* < 0.05, Fig. 1b). Moreover, the liver color turned yellow, and the liver increased in size, indicating the occurrence of fatty liver (Fig. 1c). Furthermore, the serum levels of ALT (*P* < 0.05), AST (*P* < 0.05), LDL-C (*P* < 0.05), and TC (*P* < 0.01) were increased in the HFD group (Fig. 1d). In HFD group, the liver displayed a notable increase in the accumulation of lipid droplets, as evidenced by the HE staining, Oil Red O staining, and Nile Red staining (Fig. 1e). However, the Sirius Red staining and α-SMA immunofluorescence staining results suggested that a HFD for 12 weeks did not significantly induce liver fibrosis (Additional file 1: Fig. S1a, b). Together, the above results confirm that feeding HFD to mice for 12 weeks successfully induced the NAFLD model with apparent lipid metabolism disorder.

Interestingly, the serum concentration of total iron and the concentration of Fe^2+^ in liver tissues were increased (*P* < 0.01), whereas the concentration of Fe^3+^ in liver tissues was decreased (*P* < 0.001, Fig. 1f), indicating severe dysfunction of iron metabolism in NAFLD. In the livers of the HFD group mice, the expression of both FTL and FTH exhibited simultaneous reductions compared to the NC group (*P* < 0.05, Fig. 1g). Accordingly, we attempted to explore the specific causes of liver injury based on iron metabolism.

Cav-1 has been extensively investigated in dyslipidemia, and numerous studies have revealed a potential correlation between Cav-1 genetic variations and the consumption of dietary fatty acids [25, 26]. Furthermore, our previous study demonstrated that Cav-1 can inhibit liver iron metabolism dysfunction in autoimmune hepatitis [19]. Interestingly, the immunohistochemical staining results in this study showed that Cav-1 was down-regulated in the liver tissue of the HFD group (*P* < 0.05, Fig. 1h). To further confirm the expression of Cav-1, primary hepatocytes were extracted from the livers of mice and PA + OA was used to construct a NAFLD model. The appropriate concentration and time (100 μmol/L PA + 200 μmol/L OA, 24 h or 48 h) were selected according to the CCK-8 assay results, as shown in Additional file 1: Fig. S2a. The Oil Red O staining and Nile Red staining results demonstrated significant accumulation of lipids with the PA + OA treatment (Fig. 1i, Additional file 1: Fig. S2b). Also, there were decreases in the expression of both Cav-1 (24 h:* P* = 0.0343, 48 h: *P* = 0.0017) and FTH (48 h: *P* = 0.0471) in the PA + OA group (Fig. 1j, k). These results indicate that abnormal lipid and iron metabolism are associated with the development of NAFLD and Cav-1 might play a crucial role in NAFLD.

### The iron chelator DFOM alleviated liver damage by improving iron and lipid metabolism

**Fig. 2:**
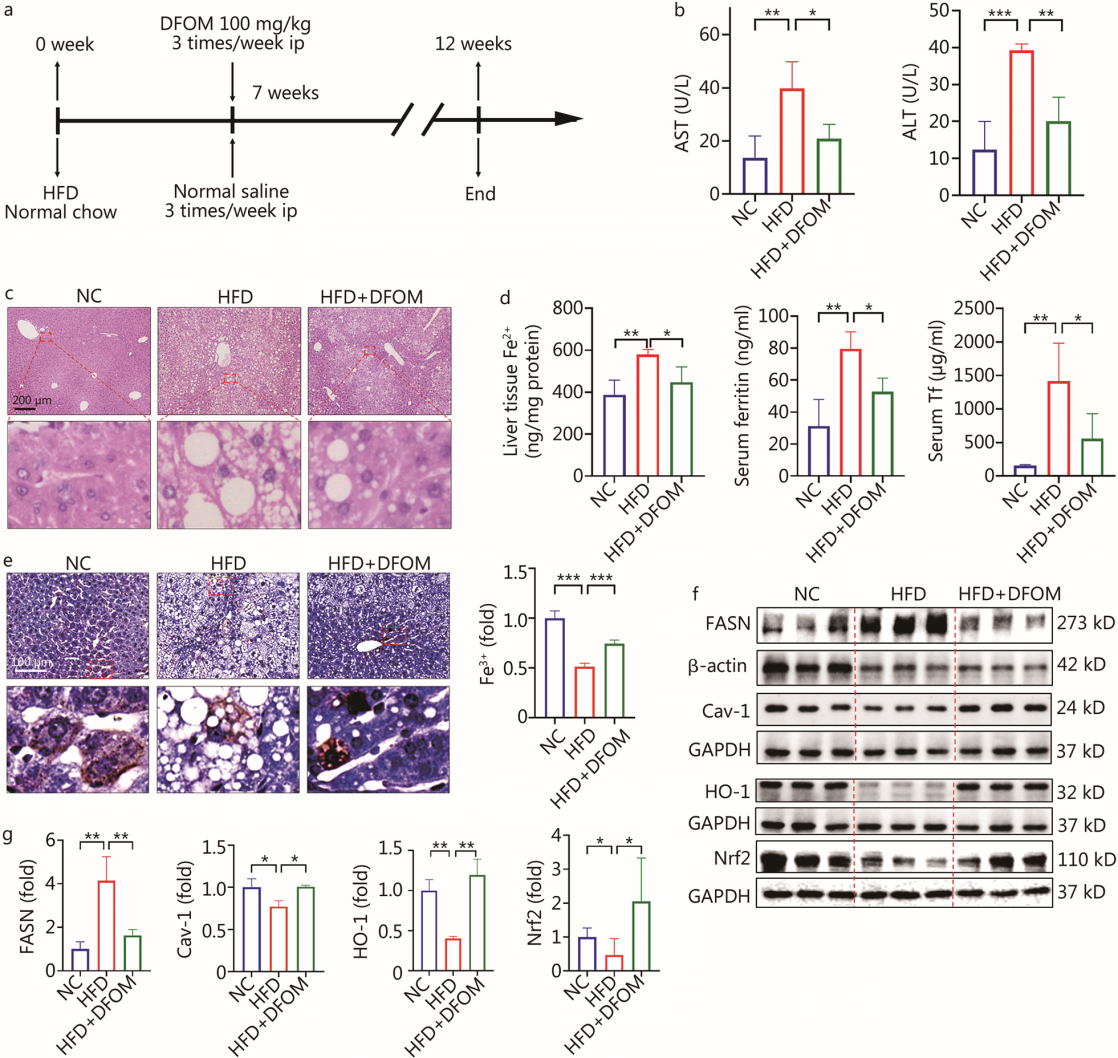
Iron chelator DFOM alleviated the development of NAFLD by improving iron and lipid metabolism. **a** Schematic diagram of DFOM intervention. **b** Serum levels of AST and ALT from mice with or without DFOM treatment (*n* ≥ 4). **c** HE staining results of liver tissue with or without DFOM treatment. **d** The concentration of liver tissue Fe^2+^, serum ferritin and serum Tf with or without DFOM treatment (*n* = 4). **e** Prussian blue staining of the liver (*n* = 4). **f**, **g** Western blotting method detected the expression of FASN, Cav-1, HO-1 and Nrf2 in liver tissue with or without DFOM treatment (*n* = 3). ^*^P < 0.05, ^**^P < 0.01, ^***^P < 0.001, as determined by one-way ANOVA. All data were shown as the mean ± SD. ns non-significant, DFOM deferoxamine mesylate, NAFLD non-alcoholic fatty liver disease, AST aspartate transaminase, ALT alanine aminotransferase, HFD high-fat diet, Tf transferrin, FASN fatty acid synthase, Cav-1 caveolin-1, Nrf2 nuclear factor erythroid 2-related factor 2, HO-1 heme oxygenase-1

Iron overload has been shown to induce cell damage and death [27]. In our study, mice were treated with or without the iron chelator DFOM (100 mg/kg) three times per week from the 7th week (Fig. 2a). As expected, serum AST and ALT detection indicated that the liver function of the HFD + DFOM group was improved compared with the HFD group (Fig. 2b). HE staining showed that the DFOM intervention reduced lipid accumulation in the liver (Fig. 2c). DFOM effectively reduced the concentration of Fe^2+^ in liver tissue, and the serum concentrations of ferritin and Tf (*P* < 0.05, Fig. 2d). In addition, a Prussian Blue Iron Stain Kit (enhanced with DAB) was used to detect the concentration of Fe^3+^. The results showed that Fe^3+^ was decreased in the HFD group (*P* < 0.001), while DFOM reversed this result (*P* < 0.001, Fig. 2e). Further, DFOM increased the expression levels of Cav-1 (*P* < 0.05), HO-1 (*P* < 0.01), and Nrf2 (*P* < 0.05), but reduced the expression of FASN (*P* < 0.01) in the liver (Fig. 2f, g). In summary, the development of NAFLD was effectively alleviated by improving iron metabolism.

### Cav-1 promoted the formation of Fe^3+^ by regulating FTL/FTH in liver tissue under high-iron status

**Fig. 3:**
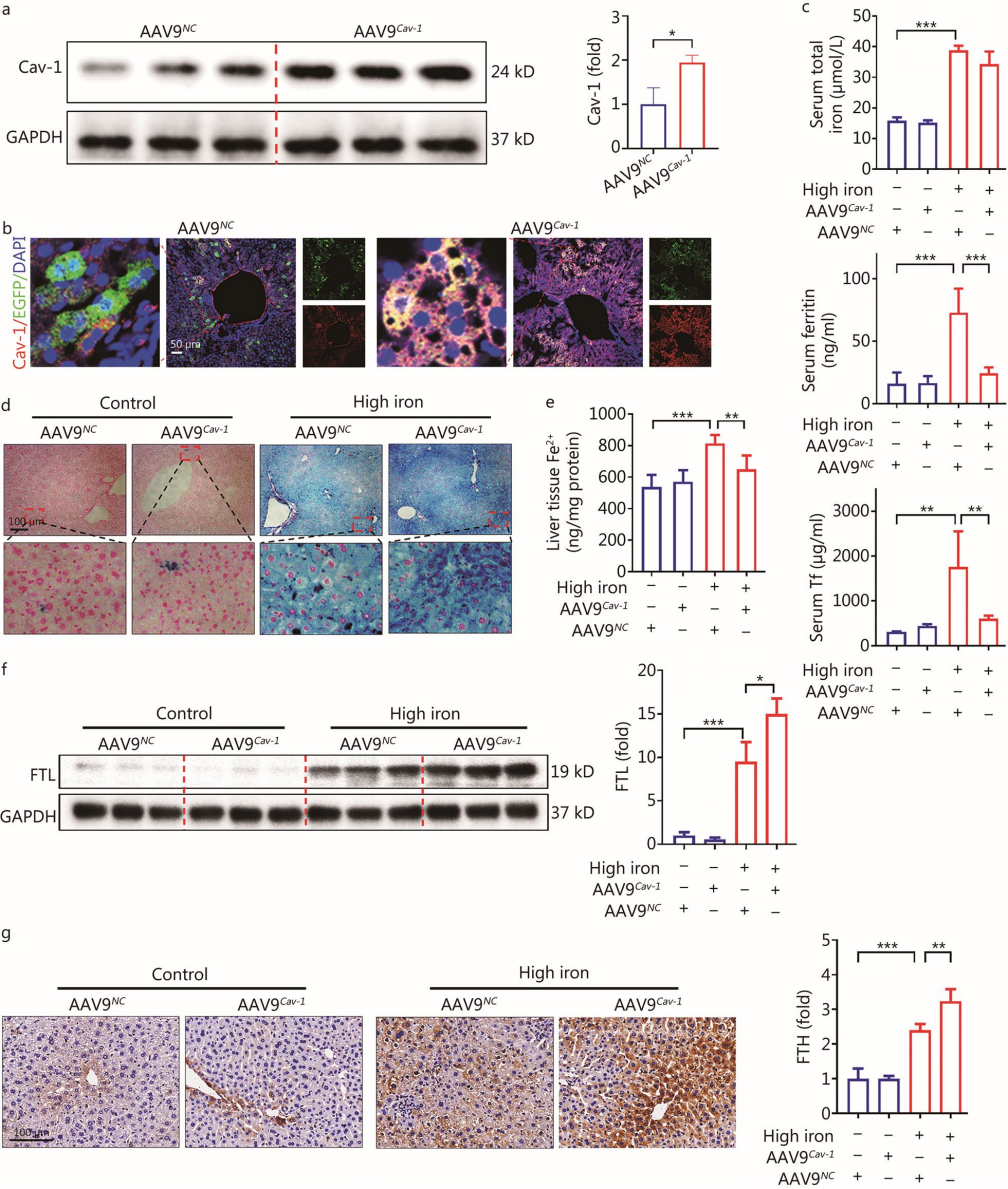
Cav-1 promoted the storage of iron in the cells as Fe^3+^. **a** Western blotting detected the expression of Cav-1 between AAV9^*NC*^ and AAV9^*Cav-1*^ (*n* = 3). **b** Immunofluorescence staining results showed the green fluorescence (EGFP) and red fluorescence (Cav-1) of liver tissue overlapped in the AAV9^*Cav-1*^ group. The red fluorescence of hepatocytes in the AAV9^*Cav-1*^ group was significantly increased (*n* = 3). **c** Concentration of serum total iron, serum ferritin and serum Tf between AAV9^*Cav-1*^ and AAV9^*NC*^ mice with or without high-iron diet (*n* = 4). **d** Prussian blue staining results in different groups between AAV9^*Cav-1*^ and AAV9^*NC*^ mice with high-iron diet. **e** The concentration of liver tissue Fe^2+^ between AAV9^*Cav-1*^ and AAV9^*NC*^ mice with or without high-iron diet (*n* = 4). **f** Western blotting results of FTL in liver tissue between AAV9^*Cav-1*^ and AAV9^*NC*^ mice with or without high-iron diet (*n* = 3). **g** Immunohistochemical staining was used to detect the expression of FTH in liver tissue between AAV9^*Cav-1*^ and AAV9^*NC*^ mice with or without high-iron diet (*n* = 4). ^*^P < 0.05, ^**^P < 0.01, ^***^P < 0.001, as determined by Student’s *t* test analysis or one-way ANOVA. All data were shown as the mean ± SD. AAV9 adeno-associated virus 9, Cav-1 caveolin-1, Tf transferrin, FTL ferritin light chain, FTH ferritin heavy chain

AAV9 was used to successfully generate hepatocyte-specific *Cav-1* overexpression mice to explore the relationship between Cav-1 and iron metabolism. First, the Western blotting results showed that the expression of Cav-1 in the liver tissue of AAV9^*Cav-1*^ mice was increased (*P* < 0.05, Fig. 3a). AAV9 was labeled with green fluorescence. The immunofluorescence staining results revealed that the expression of Cav-1 (red) and the green fluorescence displayed overlapping signals in the liver tissue of the AAV9^*Cav-1*^ group (Fig. 3b). *Cav-1* overexpression in hepatocytes did not significantly decrease the serum total iron (*P* = 0.1061) concentration, while there were significant decreases in the serum concentrations of ferritin (*P* < 0.001) and Tf (*P* < 0.01) in the high-iron diet group (Fig. 3c). In addition, Prussian blue staining demonstrated that the overexpression of *Cav-1* in hepatocytes increased the concentration of Fe^3+^ in the high-iron model (Fig. 3d). Meanwhile, compared to AAV9^*NC*^ mice fed a high-iron diet, the concentration of Fe^2+^ in the liver tissue of AAV9^*Cav-1*^ mice fed a high-iron diet was significantly reduced (*P* < 0.01, Fig. 3e); Furthermore, the expression levels of FTL (*P* < 0.05) and FTH (*P* < 0.01) were significantly increased (Fig. 3f, g). Taken together, it appears that Cav-1 might convert Fe^2+^ to Fe^3+^, which is stored in the liver as ferritin, by regulating FTL/FTH expression.

### Overexpression of *Cav-1* in hepatocytes alleviated NAFLD progression by improving the iron storage capacity via activation of the FTL/FTH pathway

**Fig. 4:**
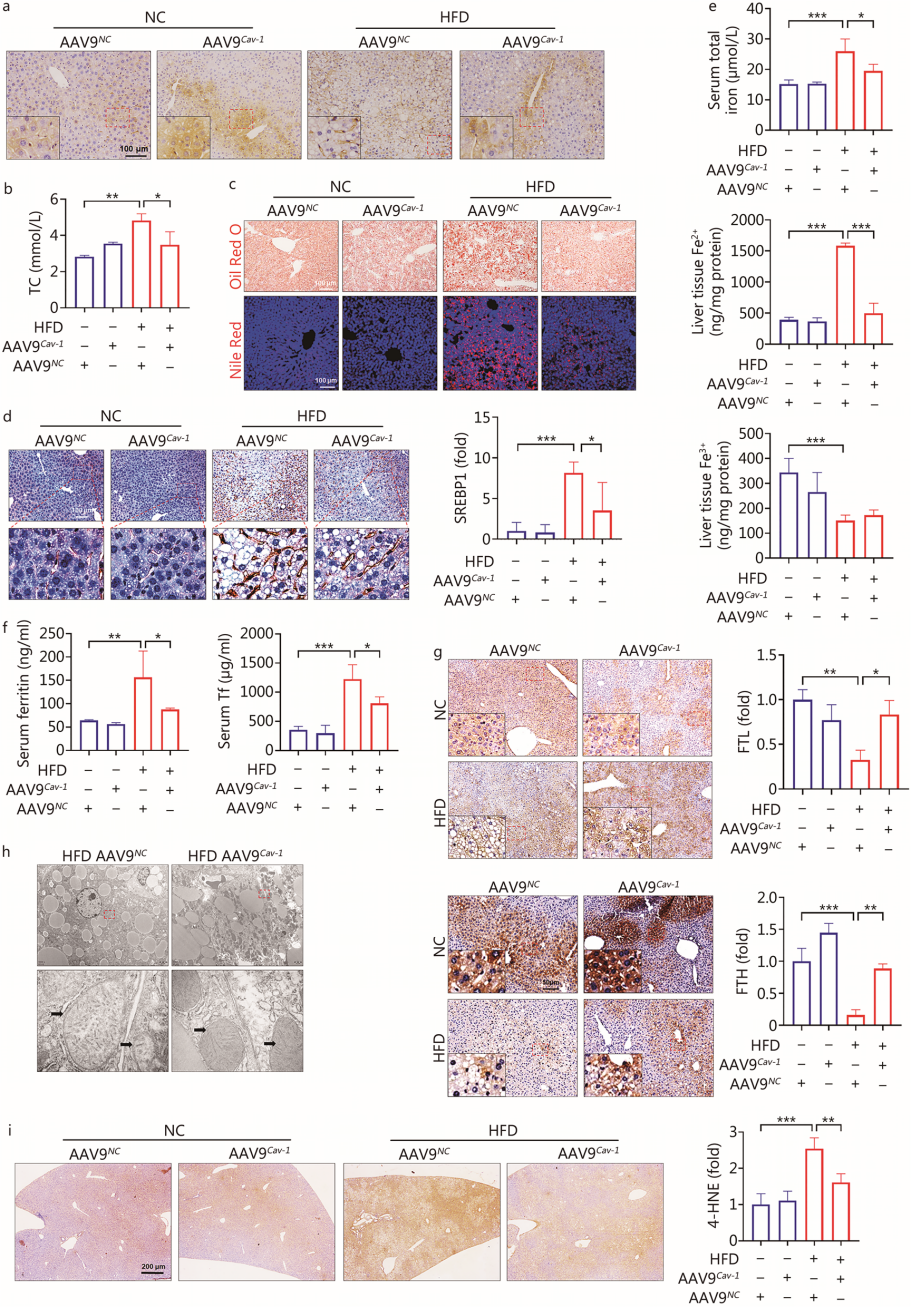
Cav-1 alleviated the development of NAFLD by up-regulating the expression of FTL/FTH. **a** Immunohistochemical staining results of Cav-1 in liver tissue between AAV9^*Cav-1*^ and AAV9^*NC*^ mice with or without high-fat diet (HFD) (*n* = 4). **b** Concentration of serum TC between AAV9^*NC*^ and AAV9^*Cav-1*^ with or without HFD (*n* = 3). **c** Oil Red O staining and Nile Red staining detected lipid accumulation in liver tissue. **d** Immunohistochemical staining detected the expression of SREBP1 protein in liver tissue (*n* = 4). **e** Concentration of serum total iron, liver tissue Fe^2+^ and liver tissue Fe^3+^ between AAV9^*Cav-1*^ and AAV9^*NC*^ mice with or without HFD (*n* = 4). **f** Concentration of serum ferritin and serum Tf in different groups (*n* = 4). **g** Immunohistochemical staining was used to detect the expression of FTL and FTH in liver tissue (*n* = 3). **h** Transmission electron microscopy to detect the changes in the mitochondrial structure of hepatocytes (*n* = 3). Black arrows indicate mitochondrion. **i** Immunohistochemical method detected the expression of 4-HNE protein in liver tissue (*n* = 4). ^*^P < 0.05, ^**^P < 0.01, ^***^P < 0.001, as determined by one-way ANOVA. All data were shown as the mean ± SD. AAV9 adeno-associated virus 9, TC total cholesterol, SREBP1 sterol regulatory element binding transcription factor 1, Tf transferrin, FTL ferritin light chain, FTH ferritin heavy chain, 4-HNE 4-hydroxynonenal

Mice with hepatocyte-specific overexpression of *Cav-1* were used to verify the role of Cav-1 in NAFLD. The immunohistochemical staining results showed that, compared with mice injected with AAV9^*NC*^, mice injected with AAV9^*Cav-1*^ had higher expression of the Cav-1 protein in the liver tissue (Fig. 4a). Overexpression of *Cav-1* inhibited the serum TC concentration (*P* < 0.05, Fig. 4b). The Oil Red O staining and Nile Red staining demonstrated that Cav-1 reduced liver lipid accumulation in NAFLD (Fig. 4c). In addition, Cav-1 inhibited the expression of SREBP1 in the liver (*P* < 0.05, Fig. 4d). Furthermore, Cav-1 inhibited the concentration of serum total iron (*P* < 0.05, Fig. 4e). Interestingly, the concentration of Fe^2+^ in liver tissue was decreased (*P* < 0.001), whereas the concentration of liver tissue Fe^3+^ was not significantly increased in the AAV9^*Cav-1*^ HFD group comparing to AAV9^*NC*^ HFD group (*P* > 0.9999, Fig. 4e). In addition, Cav-1 down-regulated the serum levels of ferritin and Tf (*P* < 0.05, Fig. 4f). To delve deeper into the precise mechanisms underlying Cav-1’s regulation of iron metabolism, additional assessments of FTL and FTH expression were performed. Compared to the AAV9^*NC*^ HFD group, the expression levels of FTL (*P* < 0.05) and FTH (*P* < 0.01) in the liver were up-regulated in the AAV9^*Cav-1*^ HFD group (Fig. 4g). These results suggested that Cav-1 decreases the accumulation of liver tissue Fe^2+^ by activating the pathway of FTL/FTH. Iron overload has been shown to induce oxidative stress, thereby damaging liver cells and organelles, such as mitochondria [28]. The results of transmission electron microscopy indicated that Cav-1 could retain the typical mitochondria shape (mitochondrion indicated by black arrows) (Fig. 4h). Furthermore, Cav-1 suppressed oxidative stress by inhibiting 4-HNE expression (*P* < 0.01, Fig. 4i). Therefore, Cav-1 plays a vital role in alleviating liver damage by improving the iron storage capacity via activation of the FTL/FTH pathway.

### Knockout of ***Cav-1*** in hepatocytes aggravated liver injury by promoting Fe^2+^ accumulation in NAFLD

**Fig. 5:**
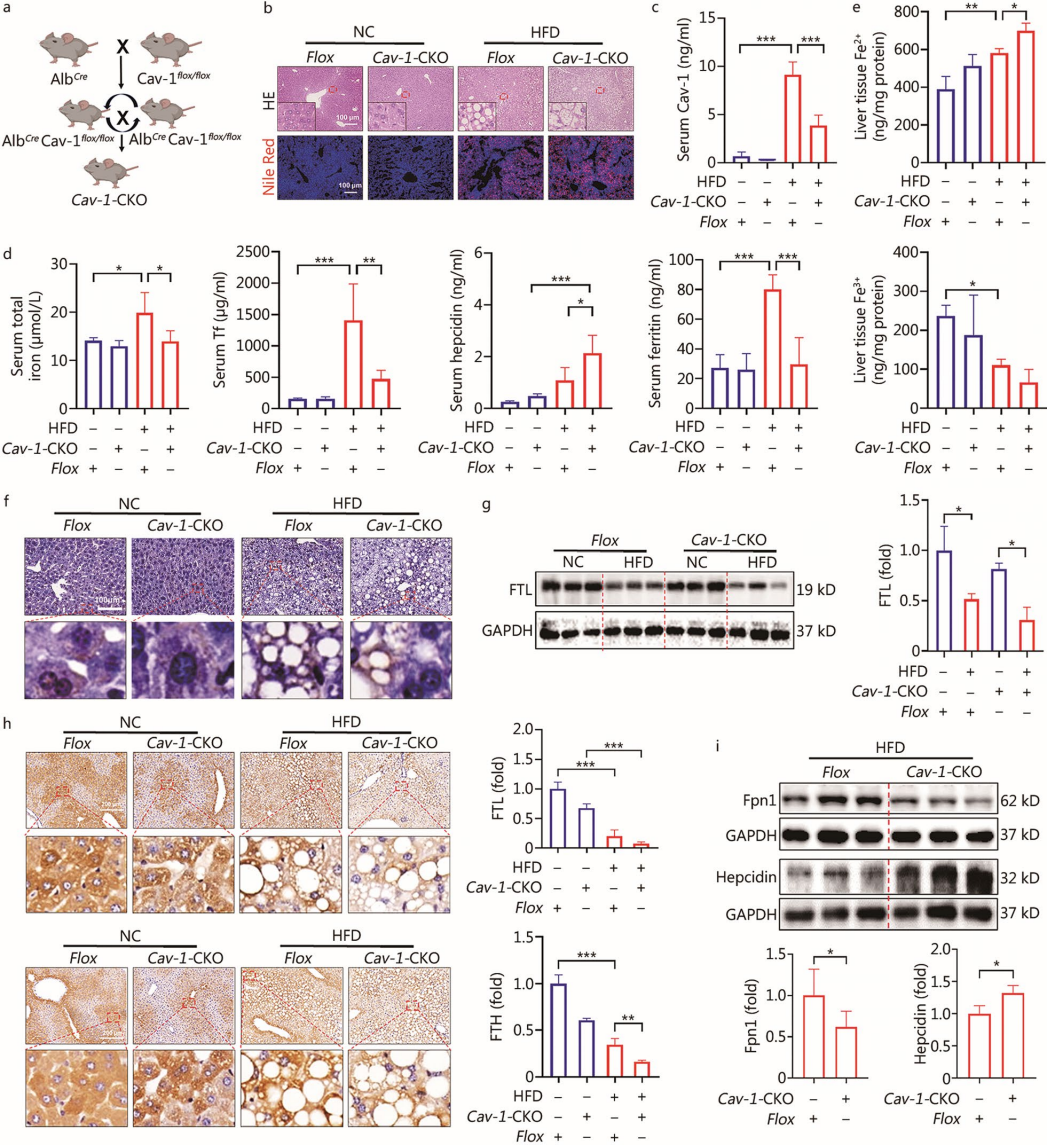
Cav-1 deficiency in hepatocytes aggravated the accumulation of liver Fe^2+^ in NAFLD. **a** Schematic construction of hepatocellular-specific *Cav-1* knockdown mice. **b** HE staining and Nile Red staining results of liver tissue. **c** The concentration of serum Cav-1 between *Flox* mice and *Cav-1*-CKO mice with or without high-fat diet (HFD) (*n* = 4). **d** The concentration of serum total iron, serum Tf, serum hepcidin, and serum ferritin in different groups (*n* = 4). **e** The concentration of Fe^2+^ and Fe^3+^ in liver tissue between *Flox* mice and *Cav-1*-CKO mice with or without HFD (*n* = 4). **f** Prussian blue staining results in different groups. **g** The expression of FTL in liver tissue was detected by Western blotting (*n* = 3). **h** Immunohistochemical method detected the expression of FTL and FTH protein in liver tissue (*n* = 4). **i** The expression of Fpn1 and hepcidin in liver tissue between *Flox* mice and *Cav-1*-CKO mice with HFD (*n* ≥ 3). ^*^P < 0.05, ^**^P < 0.01, ^***^P < 0.001, as determined by Student’s *t* test analysis or one-way ANOVA. All data were shown as the mean ± SD. *Cav-1*-CKO *Cav-1*-knockout mice, Cav-1 caveolin-1, NAFLD non-alcoholic fatty liver disease, Tf transferrin, FTL ferritin light chain, FTH ferritin heavy chain, Fpn1 ferroportin 1, PA palmitic acid, OA oleic acid

*Cav-1-*CKO mice were used to further verify the role of Cav-1 in iron metabolism from the opposite perspective (Fig. 5a; Additional file 1: Fig. S3a, b). Interestingly, knockout of *Cav-1* in hepatocytes promoted de novo lipogenesis by up-regulating the expression of FASN (*P* < 0.001, Additional file 1: Fig. S3c). However, compared with the *Flox* mice, there was no significant increase in hepatic steatosis in the *Cav-1*-CKO mice (Fig. 5b, Additional file 1: Fig. S3d). Thus, there are likely other pathways that regulate lipid metabolism. Compared to *Flox* HFD group, the serum level of Cav-1 was significantly reduced in the *Cav-1*-CKO HFD group (*P* < 0.001, Fig. 5c). In addition, knockout of *Cav-1* aggravated liver injury in NAFLD, which was manifested by a reduced concentration of SOD in the liver tissue (*P* < 0.05, Additional file 1: Fig. S3e). Further, *Cav-1* deficiency reversed the changes in the serum total iron (*P* < 0.05), Tf (*P* < 0.01) and ferritin (*P* < 0.001) levels induced by a HFD (Fig. 5d). However, the serum level of hepcidin was significantly increased in the *Cav-1*-CKO HFD group (*P* < 0.05, Fig. 5d). Compared with the *Flox* HFD group, the concentration of Fe^2+^ in liver tissue was prominently elevated in *Cav-1*-CKO HFD group (*P* < 0.05), whereas the level of Fe^3+^ in the liver tissue of *Cav-1*-CKO mice was not significantly decreased in the HFD group (*P* > 0.9999, Fig. 5e). The Prussian blue staining results were consistent with the above (Fig. 5f). The expression levels of FTL and FTH (*P* < 0.01) in the *Cav-1*-CKO HFD group were decreased (Fig. 5g, h). In addition, compared to the *Flox* NC group, the Western blotting results showed that the expression of Cav-1 in *Cav-1*-CKO NC group was decreased (*P* < 0.01), and the expression of Cav-1 was further inhibited by a HFD (*P* < 0.01, Additional file 1: Fig. S3b). Moreover, the expression of Fpn1 was decreased while hepcidin expression was increased in the *Cav-1*-CKO HFD group (*P* < 0.05, Fig. 5i). In summary, knockout of *Cav-1* in hepatocytes exacerbates NAFLD progression by accelerating the accumulation of Fe^2+^.

### CD68^+^CD163^+^ macrophages with expression of Cav-1 promoted liver damage by aggravating abnormal iron distribution in NAFLD

**Fig. 6:**
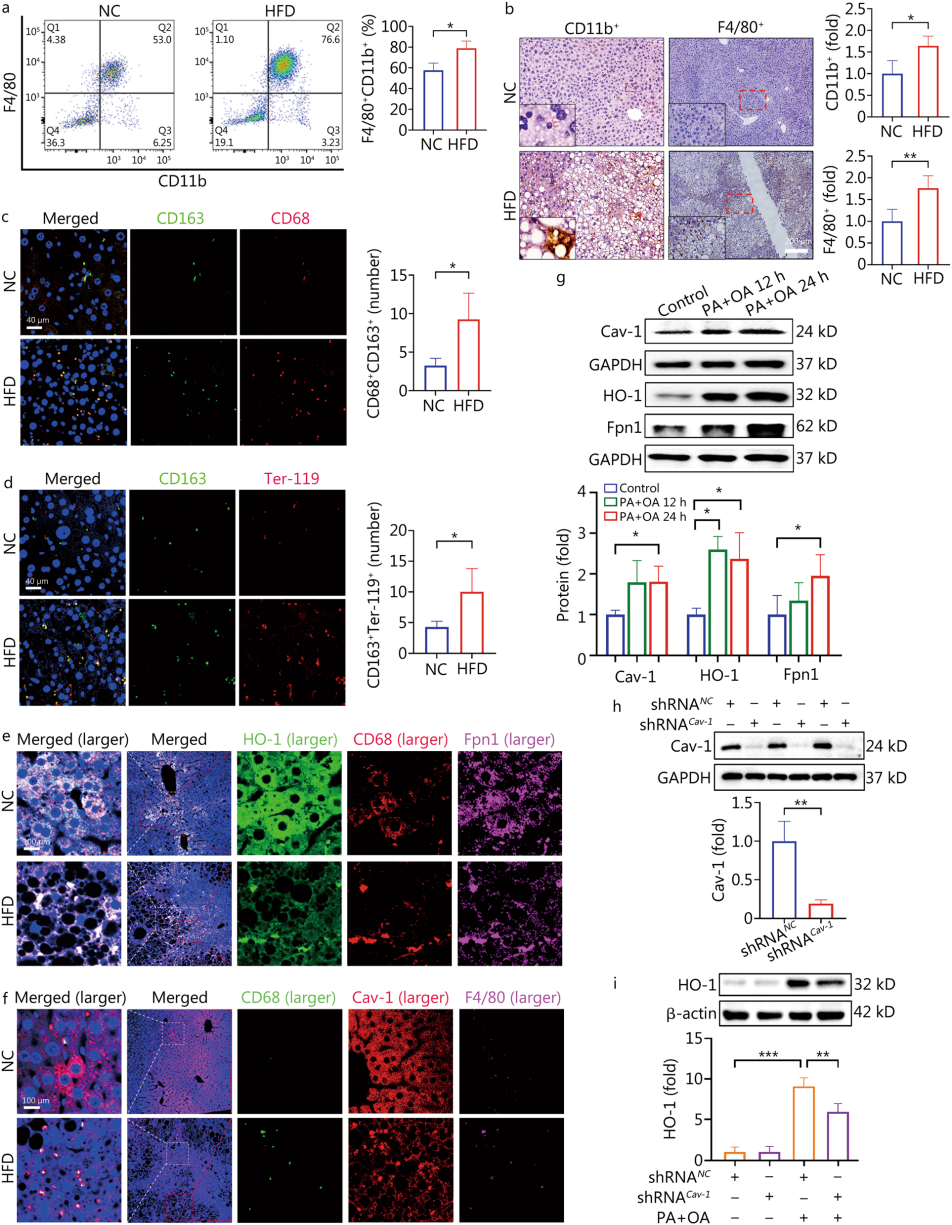
The CD68^+^CD163^+^ macrophage with high expression of Cav-1 accelerated iron homeostasis disorder in NAFLD. **a** Number of F4/80^+^CD11b^+^ macrophages in liver tissue were detected by flow cytometry (*n* = 3). **b** Expression of CD11b^+^ and F4/80^+^ in liver tissue were detected by immunohistochemical method between NC group and HFD group (*n* = 4). **c** CD68 and CD163 immunofluorescence co-staining results in liver tissue (*n* = 4). **d** CD163 and Ter-119 immunofluorescence co-staining results in liver tissue (*n* = 4). **e** Expressing of HO-1, CD68 and Fpn1 in liver tissue were detected by multiplex fluorescence immunohistochemical staining method (*n* = 3). **f** Multiplex fluorescence immunohistochemical staining method to detect the activation of F4/80^+^CD68^+^ macrophages and the expression of Cav-1 in liver tissue (*n* = 3). **g** Western blotting detected the expression of Cav-1, HO-1 and Fpn1 proteins in RAW264.7 cells with or without PA + OA (*n* ≥ 3). **h** Western blotting detected the expression of HO-1 in RAW264.7 cell between shRNA^*Cav-1*^ treatment and shRNA^*NC*^ treatment (*n* = 3). **i** Western blotting detected the expression of HO-1 in RAW264.7 cell between shRNA^*Cav-1*^ treatment and shRNA^*NC*^ treatment with or without PA + OA (*n* = 3). ^*^P < 0.05, ^**^P < 0.01, ^***^P < 0.001 as determined by Student’s *t* test analysis or one-way ANOVA. All data were shown as the mean ± SD. Cav-1 caveolin-1, NAFLD non-alcoholic fatty liver disease, HFD high-fat diet, HO-1 heme oxygenase-1, Fpn1 ferroportin 1

The number of hepatic F4/80^+^CD11b^+^ macrophages was significantly increased in the HFD group (*P* < 0.05, Fig. 6a, b). F4/80^+^/CD68^+^ represents macrophages with phagocytic capacity, and our previous study found that depletion of liver macrophages by gadolinium chloride (GdCl_3_) alleviated liver iron accumulation in autoimmune hepatitis [19]. Moreover, liver CD163^+^ macrophages promote phagocytosis and degradation of aging and damaged red blood cells to recover iron, and this plays an important role in iron metabolism [29]. Therefore, we further explored the role of iron metabolism in NAFLD from the perspective of macrophage function. CD68^+^/CD163^+^ macrophages are capable of erythrophagocytosis. Ter-119 is a confirmed red blood cell marker. The co-localization of immunofluorescence showed that compared with the control group, the number of the CD68^+^CD163^+^ and the CD163^+^ macrophages containing red blood cells (Ter-119) were both increased significantly in the HFD group (*P* < 0.05, Fig. 6c, d). In addition, HO-1 functions to decompose heme to obtain iron. Our results revealed that CD68^+^HO-1^+^ macrophages overexpressed Fpn1 (Fig. 6e). This implies that the iron decomposed in the macrophages would be subsequently excreted. Intriguingly, a decrease in Cav-1 expression was observed within hepatocytes, while an increase was noted in macrophages in NAFLD in vivo. Meanwhile, F4/80^+^CD68^+^ macrophages expressing Cav-1 were increased in the HFD group (Fig. 6f). This suggests that Cav-1 might participate in the iron metabolism of macrophages.

Consistent with the results of the in vivo experiments, treatment with PA + OA for 24 h promoted the expression of Cav-1, HO-1, and Fpn1 in RAW264.7 cells (*P* < 0.05, Fig. 6g). In order to further confirm the role of Cav-1 in macrophage iron metabolism function, *Cav-1*-knockdown RAW264.7 cells were constructed by lentivirus (Fig. 6h). *Cav-1* knockdown effectively inhibited the expression of HO-1 in macrophages treated with PA + OA for 24 h (*P* = 0.0096, Fig. 6i). In summary, HFD or PA + OA aggravated macrophage iron metabolism disorder by up-regulating the expression of Cav-1 and HO-1.

### Serum Cav-1 served as a key indicator to monitor iron homeostasis in patients with NAFLD

**Fig. 7:**
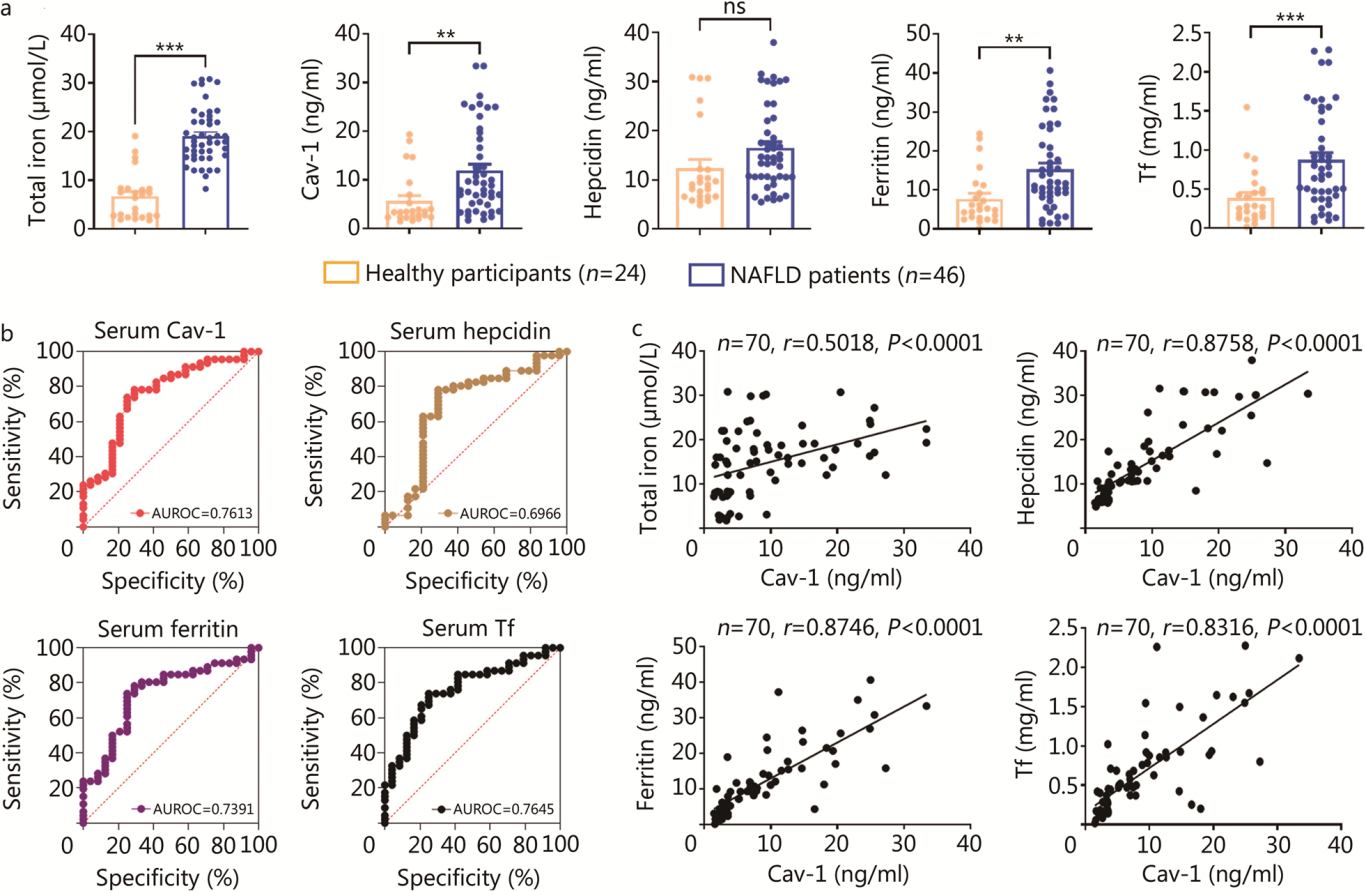
Serum Cav-1 was a pivotal indicator in NAFLD and positively correlated with serum iron-related proteins in volunteers. **a** Detection of serum total iron, Cav-1, hepcidin, ferritin and Tf concentrations in healthy people (*n* = 24, the same below) and NAFLD patients (*n* = 46, the same below). **b** The AUROC analysis of serum Cav-1, hepcidin, ferritin and Tf levels. **c** Correlation analysis of serum Cav-1 concentration and serum of total iron, hepcidin, ferritin, and Tf concentration. ^**^P < 0.01, ^***^P < 0.001, as determined by Student’s *t* test analysis, Spearman’s rank correlation test or receiver operating characteristic curve analysis. All data were shown as the mean ± SD. AUROC area under the receiver operating characteristic curve, Cav-1 caveolin-1, Tf transferrin, NAFLD non-alcoholic fatty liver disease

A total of 52 patients with NAFLD participated in this study; six were excluded as they were positive for hepatitis B. In addition, 29 healthy volunteers were recruited to participate in this study; five were excluded (three had consumed alcohol in the past two weeks and two had a history of diabetes, Additional file 1: Fig. S4). Compared with the healthy participants, the serum concentrations of total iron (*P* < 0.001), Cav-1 (*P* < 0.01), ferritin (*P* < 0.01), and Tf (*P* < 0.001) were up-regulated in the patients with NAFLD; however, the concentration of hepcidin (*P* = 0.0598) was not significantly increased (Fig. 7a). The Cav-1, hepcidin, ferritin, and Tf concentrations could individually highlight the differences between the healthy participants and patients with NAFLD in the high AUROCs (Fig. 7b). The AUROC of the serum Cav-1 protein concentration (AUROC = 0.7613, *P* = 0.0004) was higher than that of the other serum proteins, including hepcidin (AUROC = 0.6966,* P* = 0.0073) and ferritin (AUROC = 0.7391,* P* = 0.0011), but lower than that of Tf (AUROC = 0.7645, *P* = 0.0003, Additional file 1: Table S3). To control for potential confounding factors that may influence the correlation analysis between Cav-1 and iron metabolism indicators, multivariate linear regression analysis was used to examine the relationship between Cav-1 and multiple intervention factors. Hepcidin was significantly associated with the level of Cav-1 (*P* < 0.001), while BMI, ALT, and other measures were not significantly associated with Cav-1 (Additional file 1: Table S4). Likewise, ferritin (*P* = 0.0007), Tf (*P* = 0.0003), and iron (*P* = 0.0513, approximately 0.05) were significantly associated with Cav-1, respectively (Additional file 1: Tables S5–S7). Furthermore, there were clear positive correlations between Cav-1 and several iron-related proteins, such as hepcidin (*r* = 0.8758, *P* < 0.001), ferritin (*r* = 0.8746, *P* < 0.001), and Tf (*r* = 0.8316, *P* < 0.001, Fig. 7c). Therefore, Cav-1 might serve as a clinical diagnostic indicator for NAFLD iron metabolism homeostasis.

## Discussion

**Fig. 8:**
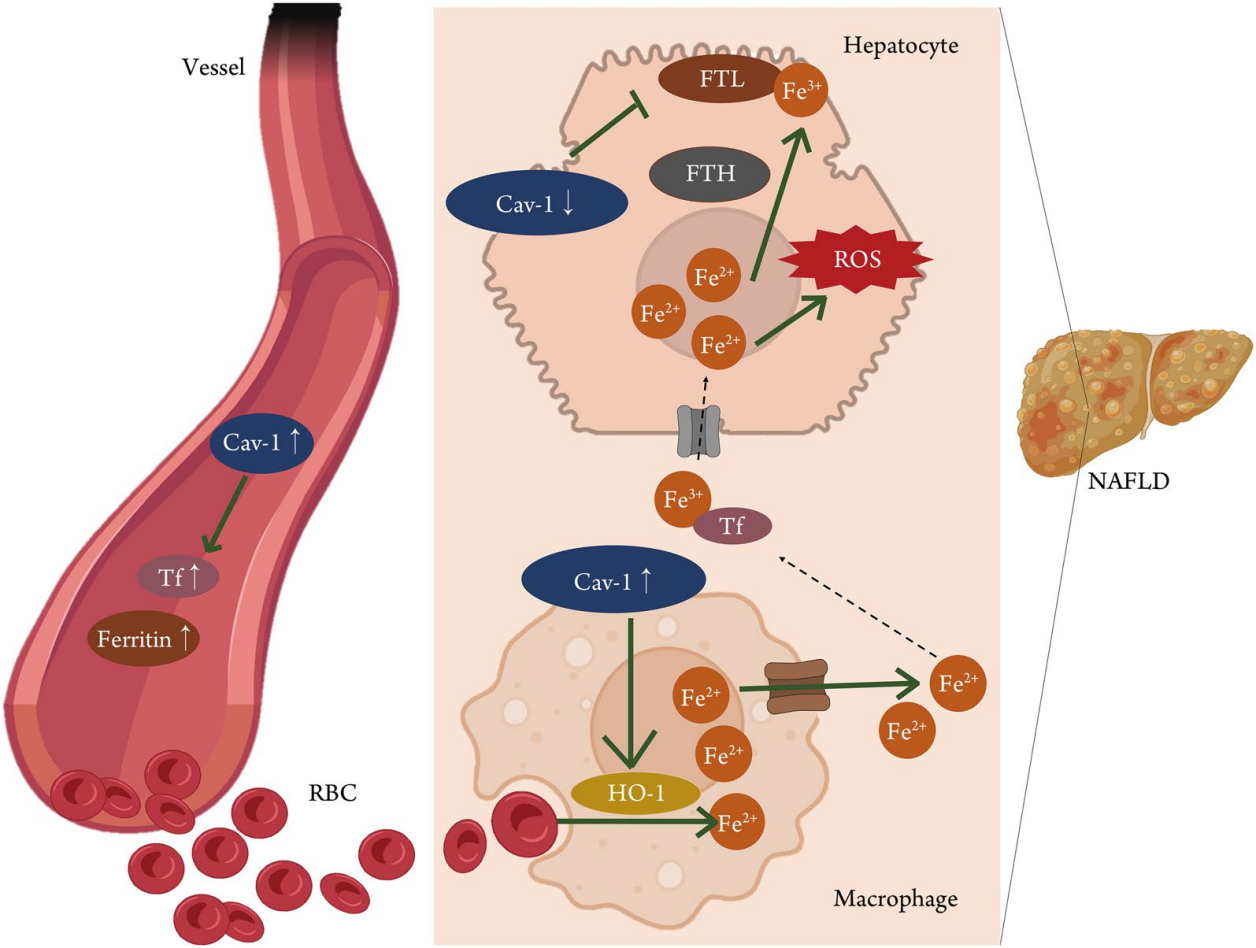
Scheme of Cav-1 inhibiting the development of NAFLD by regulating iron metabolism. The HFD inhibited the expression of Cav-1 in hepatocytes and up-regulated the expression of Cav-1 in liver macrophages. In hepatocytes, Cav-1 regulated the FTL/FTH signaling pathway to promote the conversion of Fe^2+^ to Fe^3+^, which helped to slow down the oxidative stress induced by Fe^2+^ and ultimately alleviate the progression of NAFLD. In macrophages, Cav-1 regulated the expression of HO-1 in CD68^+^CD163^+^ macrophages, and promoted the degradation of red blood cells into Fe^2+^ within macrophages which ultimately exacerbated the disorder of iron metabolism in the liver. In addition, the concentration of ferritin and transferrin in serum was up-regulated by Cav-1, and the two were positively correlated in human subject. HFD high-fat diet, Cav-1 caveolin-1, Tf transferrin, NAFLD non-alcoholic fatty liver disease, HO-1 heme oxygenase-1, FTL ferritin light chain, FTH ferritin heavy chain, RBC red blood cell, ROS reactive oxygen species

In this study, we found that the HFD not only inhibits the expression of Cav-1 in hepatocytes but also up-regulates the expression of Cav-1 in macrophages. Cav-1 down-regulation in hepatocytes inhibited the process that converse the Fe^2+^ to Fe^3+^ through the regulation of the FTL/FTH signaling pathway, leading to the accumulation of Fe^2+^ in hepatocytes which aggravated the oxidative stress induced by Fe^2+^ and ultimately accelerated the progression of NAFLD. In macrophages, the up-regulation of Cav-1 promoted the expression of HO-1 in CD68^+^CD163^+^ macrophages, which facilitated the degradation of red blood cells into Fe^2+^ within macrophages and accelerated iron metabolism disorder in NAFLD. In addition, the concentration of ferritin and transferrin in serum was up-regulated with the increase of  serum Cav-1 concentration, and the two were positively correlated respectively (Fig. 8).

NAFLD is a metabolic disorder characterized by abnormal lipid metabolism [30]. It encompasses various histopathological alterations, ranging from simple steatosis to steatohepatitis with or without fibrosis [31], and ultimately progresses to liver cirrhosis or hepatocellular carcinoma [32]. Scientists have proposed the “multiple-hit” mechanistic theory of NAFLD [33]. This is based on the knowledge that lipid metabolism disorder induces cell damage, which is directly or indirectly caused by iron overload, oxidative stress, inflammatory cell activation, or other pathological factors [34]. Hence, this study explored the molecular mechanisms underlying the occurrence and development of NAFLD mainly from the perspective of iron metabolism disorder.

Cav-1 is an integral membrane protein and is widely distributed in endothelial cells, fibroblasts, adipocytes, hepatocytes, and macrophages [35, 36]. It has been reported that Cav-1 plays a pivotal role in the development of NAFLD [37] due to its roles in lipid metabolism, cholesterol transport [38], and membrane structural stability. Recently, Cav-1 has been widely studied in dyslipidemia. One study found that a Cav-1 genetic polymorphism interacted with polyunsaturated fatty acids to modulate metabolic syndrome risk [25]. However, the role of Cav-1 in NAFLD remains controversial. Thus, it was necessary to further reveal the specific mechanism of Cav-1 in NAFLD. To this end, an in vivo NAFLD model was constructed by feeding a HFD for 12 weeks. Then, PA + OA was used to induce the NAFLD model in primary hepatocytes in vitro. Interestingly, HFD or PA + OA decreased the expression of Cav-1 in vivo and in vitro. Moreover, hepatocyte-specific *Cav-1* overexpression or knockout mice were used to study whether Cav-1 might inhibit de novo lipogenesis by down-regulating the expression of FASN [39]. Consistent with previous studies, compared to *Flox* mice, hepatocyte-specific *Cav-1* knockout mice did not exhibit significantly increased liver lipid accumulation [36]. This suggests that Cav-1 might affect other lipid metabolic pathways. There is ongoing controversy about the roles of Cav-1 in NAFLD, which might be related to modeling methods or mouse strains [40]; thus, it remains necessary to comprehensively analyze the roles of Cav-1 and reveal its biological functions in NAFLD.

Recent research has revealed a potential association between iron overload and the pathogenesis of NAFLD [6], as well as iron deficiency [7]. However, iron overload confers greater susceptibility to the development of fatty liver disease [8]. Therefore, lipid and iron metabolism may have a close relationship. Excess iron could promote the generation of toxic ROS through the Fenton reaction [41], further leading to hepatocyte damage and death, and ultimately triggering the development of NAFLD [10]. The pharmacological inhibition of iron effectively alleviates the development of NAFLD [42]. Consistent with our previous report, the current in vivo experiments confirmed that Fe^3+^ was decreased and Fe^2+^ was increased in NAFLD. Ferritin consists of two distinct subunit types, H (heavy) and L (light) [43]. FTH mainly oxidizes Fe^2+^ to Fe^3+^ [44], and then FTH forms a complex with FTL to enclose Fe^3+^ as a form of iron storage. Interestingly, the expression levels of FTL and FTH were down-regulated in NAFLD, which could explain the increased Fe^2+^. These findings suggest that disrupted iron metabolism plays a role in the development of NAFLD. To further clarify the role of iron in the development of NAFLD, the iron chelator DFOM was intraperitoneally injected into the mice. DFOM was found to alleviate NAFLD development by inhibiting excessive iron accumulation. Interestingly, DFOM also inhibited the expression of FASN and promoted the expression of Cav-1, indicating that DFOM can suppress the development of NAFLD by regulating the expression of FASN and Cav-1. Nrf2 is a regulator of cellular resistance to oxidants. Nrf2 exists in the cytoplasm in an inactive state bound to the Keap1 protein, which is degraded by targeted proteases to maintain the low transcriptional activity of Nrf2. However, the Nrf2-Keap1 interaction is dissociated in a dose-dependent manner under oxidative stress [45]. In addition, the Nrf2/HO-1 signaling pathway is a pivotal signaling pathway involved in sensing the environment and regulating endogenous oxidative stress; it maintains cell redox homeostasis by inducing protective genes through transcription [46]. Interestingly, the current results revealed that DFOM up-regulated the expression of Nrf2 and HO-1 in liver tissue. Therefore, DFOM inhibited the oxidative stress response induced by iron accumulation by up-regulating the expression of Nrf2 and HO-1.

Our previous study found that Cav-1 alleviated liver injury in autoimmune hepatitis by inhibiting iron accumulation in the liver [19]. According to our study results, we speculated that Cav-1 plays a pivotal role in the regulation of iron metabolism in the liver. Thus, we constructed a high-iron model by feeding a high-iron diet for 12 weeks in vivo. Interestingly, we found that hepatocyte-specific *Cav-1* overexpression decreased the level of Fe^2+^ by activating the FTL/FTH pathway, which might inhibit liver injury. Based on the above results, we utilized hepatocyte-specific *Cav-1* overexpression or knockout mice to further explore the iron regulating role of Cav-1 in NAFLD. The results demonstrated that Cav-1 reduced the concentrations of Fe^2+^ in the liver tissue to alleviate the development of NAFLD by up-regulating the FTL/FTH pathway. However, compared to *Flox* HFD group, the serum total iron, serum ferritin, and serum Tf concentrations were down-regulated in the *Cav-1*-CKO HFD group. This seemed to contradict the previous conclusion that Cav-1 inhibits iron overload. Interestingly, Cav-1 inhibited the expression of hepcidin and activated the expression of Fpn1, which suggests that Cav-1 down-regulate serum iron levels by regulating iron transport. Therefore, knockout of *Cav-1* not only promoted Fe^2+^ accumulation by inhibiting the expression of FTL and FTH, but also down-regulated the serum concentrations of total iron and iron-related proteins by decreasing the expression of Fpn1. Taken together, these data suggest that Cav-1 alleviates iron accumulation by improving the iron storage capacity and transport capacity of hepatocytes, thereby alleviating the oxidative stress induced by Fe^2+^.

CD11b^+^F4/80^high^ Kupffer cells exhibit a less inflammatory phenotype than CD11b^+^F4/80^low^ macrophages in the livers of HFD-fed WT mice [47]. The current results revealed that a HFD induced an increase in macrophages in the F4/80^+^CD11b^+^ group. Our previous study confirmed that GdCl_3_ depleted liver macrophages can alleviate the progression of autoimmune hepatitis by reducing the concentration of iron [19]. Therefore, macrophages might play an important role in the development of NAFLD. A significant increase in F4/80^+^CD11b^+^CD68^+^ macrophages can accelerate the progression of NAFLD [48]. CD163^+^ macrophages phagocytize senescent or damaged red blood cells [49]. Research has shown that CD163^+^ macrophages might induce liver iron overload due to their potent phagocytic activity and clearance of red blood cells in a pathological state [50]. In addition, the expression of HO-1 plays an important role in the normal function of CD163 phagocytosis and the metabolism of aged erythrocytes [51]. Therefore, to further explore the source of hepatic iron in NAFLD, we analyzed the correlation between macrophage function and the hepatic iron overload microenvironment. We hypothesized that CD68^+^CD163^+^ macrophages are infiltrated and activated in NAFLD, thereby increasing the phagocytosis of senescent or damaged red blood cells [52]. HO-1 expressed in CD68^+^ macrophages decompose hemoglobin into Fe^2+^ [53], which is eventually excreted outside the macrophage and forms an iron-overloaded microenvironment in the liver [54]. As expected, in this study, CD68^+^ macrophages in the liver tissue expressed high levels of Cav-1, HO-1, and Fpn1. We co-stained liver tissues to detect CD68 and CD163 and verified the speculation that CD68^+^CD163^+^ macrophages phagocytize and decompose erythrocytes to produce excessive Fe^2+^, thus exacerbating iron overload in the liver. In addition, the in vitro experimental results further confirmed that Cav-1 promotes the expression of HO-1. In conclusion, the disturbances in hepatic iron metabolism are not only related to the iron storage capacity of hepatocytes but also to the erythrocyte phagocytic function of CD68^+^CD163^+^ macrophages.

To further clarify the clinical significance of Cav-1, we analyzed the correlations between the serum Cav-1 level and iron metabolism-related proteins, such as hepcidin, ferritin, and Tf. A total of 46 patients and 24 healthy volunteers were included in this analysis. Previous studies have demonstrated that as a marker of liver iron storage, serum ferritin might be a dynamic indicator of NAFLD progression [55]. Hepcidin is mainly synthesized and secreted in the liver and is highly expressed in NAFLD patients. Our study found that compared to healthy volunteers, the serum Cav-1 concentration was significantly increased in patients with NAFLD, and significant positive correlations were observed between the serum Cav-1 concentration and the serum ferritin, hepcidin, and Tf concentrations. In summary, these results indicate that the serum Cav-1 concentration could serve as a novel and reliable clinical indicator for monitoring the iron metabolism homeostasis of NAFLD patients. However, whether dynamic changes in the serum Cav-1 level occur at different stages of NAFLD remains to be further explored.

## Conclusions

These findings indicate that Cav-1 is an essential target protein that regulates lipid metabolic homeostasis, and regulates iron metabolic homeostasis by activating FTL/FTH pathway. The serum level of Cav-1 could serve as an indicator for monitoring the development of NAFLD. Additionally, hepatic macrophages regulate iron metabolism through the Cav-1/HO-1 pathway, contributing to the progression of NAFLD. Overall, this study demonstrates that Cav-1 is a pivotal molecule for predicting and protecting against the development of NAFLD.

### Supplementary Information

**Additional file 1: Table S1.** Sequences in Cav-1^*flox/flox*^ and Alb^Cre^ mice construction. **Table S2.** Sequences in shRNA^*Cav-1*^ and shRNA^*NC*^ cells construction. **Table S3.** AUROC of four serum proteins in 70 volunteers (*n* = 70). **Table S4.** Coefficients between Cav-1 and covariates (hepcidin and confounders) in MLR model. **Table S5.** Coefficients between Cav-1 and covariates (ferritin and confounders) in MLR model. **Table S6.** Coefficients between Cav-1 and covariates (transferrin and confounders) in MLR model. **Table S7.** Coefficients between Cav-1 and covariates (iron and confounders) in MLR model. **Fig. S1.** Changes of liver fibrosis in mice after 12 weeks of high-fat diet (HFD). **Fig. S2.** PA + OA successfully constructed primary hepatocytes NAFLD model. **Fig. S3.** Hepatocyte-specific *Cav-1* knockdown affected the development of NAFLD. **Fig. S4.** Flow diagram of participants enrolled in the two study groups.

## Data Availability

All data generated or analysed during this study are included in this published article.

## Abbreviations

AAV9^*Cav1*^: *Cav-1* overexpression adeno-associated virus
AAV9^*NC*^: Negative control adeno-associated virus
AST: Aspartate transaminase
ALT: Alanine aminotransferase
ALB: Albumin
α-SMA: Alpha-smooth muscle actin
AUROC: Area under the receiver ooperating characteristic curve
BMI: Body mass index
BSA: Bovine serum albumin
*Cav-1*-CKO: *Cav-1*-knockout mice
Cav-1: Caveolin-1
Cr: Creatinine
DFOM: Deferoxamine mesylate
DAB: Diaminobenzidine
*Flox*: Cav-1^*flox/flox*^
FBS: Fetal bovine serum
FFA: Free fatty acids
FTL: Ferritin light chain
FTH: Ferritin heavy chain
Fe^2+^: Ferrous irons
Fe^3+^: Ferric irons
Fpn1: Ferroportin 1
FASN: Fatty acid synthase
FACS: Fluorescence-activated cell sorting
FBG: Fasting blood-glucose
GdCl_3_: Gadolinium chloride
γ-GGT: γ-Glutamine
HFD: High-fat diet
HO-1: Heme oxygenase-1
4-HNE: 4-Hydroxynonenal
HNPC: Hepatic non-parenchymal cells
LDL-C: Low-density lipoprotein-cholesterol
NAFLD: Nonalcoholic fatty liver disease
Nrf2: Nuclear factor erythroid 2-related factor 2
NC: Normal diet
OA: Oleic acid
OCT: Optimal cutting temperature compound
PVDF: Polyvinylidene fluoride membranes
PA: Palmitic acid
ROS: Reactive oxygen species
RT: Room temperature
SOD: Superoxide dismutase
SD: Standard deviation
shRNA^*Cav-1*^: *Cav-1* knockdown
SREBP1: Sterol regulatory element binding transcription factor 1
Tf: Transferrin
TC: Total cholesterol
TG: Triglyceride
TBiL: Total bilirubin
UA: Uric acid
WT: Wild-type
