# Supplementary material for: Caveolin-1 is critical for hepatic iron storage capacity in the development of nonalcoholic fatty liver disease
Source: Mil Med Res. 2023 Nov 8;10:53. doi: 10.1186/s40779-023-00487-3 (PMC10631186; doi:10.1186/s40779-023-00487-3)
Supplement: Supplementary file 1 — Additional file 1: Table S1. Sequences in Cav-1flox/flox and AlbCre mice construction. Table S2. Sequences in shRNACav-1 and shRNANC cells construction. Table S3. AUROC of four serum proteins in 70 volunteers (n = 70). Table S4. Coefficients between Cav-1 and covariates (hepcidin and confounders) in MLR model. Table S5. Coefficients between Cav-1 and covariates (ferritin and confounders) in MLR model. Table S6. Coefficients between Cav-1 and covariates (transferrin and confounders) in MLR model. Table S7. Coefficients between Cav-1 and covariates (iron and confounders) in MLR model. Fig. S1. Changes of liver fibrosis in mice after 12 weeks of high-fat diet (HFD). Fig. S2. PA + OA successfully constructed primary hepatocytes NAFLD model. Fig. S3. Hepatocyte-specific Cav-1 knockdown affected the development of NAFLD. Fig. S4. Flow diagram of participants enrolled in the two study groups. [file 40779_2023_487_MOESM1_ESM.pdf]

Table S1 Sequences in Cav-1$^{flox/flox}$ and Alb^{Cre} mice construction

**Name**	**Sequence (5'–3')**
Cav-1$^{flox/flox}$	F: TCGCAGTGATGATAACTCCATGA
	R: TGAGGACTACTGCTCAGGTTAAAT
Alb^{Cre}	M-F: GAAGCAGAAGCTTAGGAAGATGG
	M-R: TTGGCCCCTTACCATAACTG
	W-F: TGCAAACATCACATGCACAC
loxP	F: TCGCAGTGATGATAACTCCATGA
	R: TGAGGACTACTGCTCAGGTTAAAT

F forward, *R* reverse

Table S2 Sequences in shRNA^{Cav-1} and shRNA^{NC} cells construction

**Name**	**Sequence (5'–3')**
shRNA^{Cav-1}	CCACCTTCACTGTGACGAAAT
shRNA^{NC}	TTCTCCGAACGTGTCACGT

Table S3 AUROC of four serum proteins in 70 volunteers (n = 70)

**Serum indexes**	**AUROC**	**95%CI**	***P* value**
Cav-1	0.7613	0.6406 – 0.8820	0.0004
Hepcidin	0.6966	0.5539 – 0.8393	0.0073
Ferritin	0.7391	0.6162 – 0.8621	0.0011
TF	0.7645	0.6496 – 0.8793	0.0003

AUROC area under the receiver operating characteristic curve, *Cav-1* caveolin-1, *TF* transferrin

Table S4 Coefficients between Cav-1 and covariates (hepcidin and confounders) in MLR model

**Name**	**Estimate**	**Std. Error**	***t* value**	**Pr (>|*t*|)**	**Significant**
Intercept	-5.92252	6.854686	-0.86401	0.391268	
Hepcidin	0.402134	0.113642	3.538591	0.000817	***
BMI	-0.21502	0.258683	-0.83122	0.409378	
ALT	0.151294	0.085962	1.760015	0.083866	
AST	-0.00267	0.038557	-0.06933	0.944973	
γ-GGT	-0.03731	0.057416	-0.6499	0.518413	
TBiL	-0.0197	0.113962	-0.17285	0.863392	
ALB	0.281823	0.238381	1.182238	0.242107	
Cr	-0.03778	0.085135	-0.44382	0.65888	
UA	-0.00809	0.017582	-0.46012	0.647213	
TG	0.711968	1.947806	0.365523	0.716098	
TC	-1.39937	2.214105	-0.63203	0.529942	
LDL-C	0.542566	2.428263	0.223438	0.824008	
FBG	2.23873	1.433228	1.562019	0.123917	

$^{***}P < 0.001$; *Cav-1* caveolin-1, *BMI* body mass index, *ALT* alanine aminotransferase, *AST* aspartate transaminase, *γ-GGT* γ-glutamine, *TBiL* total bilirubin, *ALB* albumin, *Cr* creatinine, *UA* uric acid, *TG* triglyceride, *TC* total cholesterol, *LDL-C* low-density lipoprotein-cholesterol, *FBG* fasting blood-glucose, *MLR* multiple linear regression

Table S5 Coefficients between Cav-1 and covariates (ferritin and confounders) in MLR model

**Name**	**Estimate**	**Std. Error**	***t* value**	**Pr (>|*t*|)**	**Significant**
Intercept	-3.17852	6.851	-0.46395	0.644482	
Ferritin	0.353773	0.098825	3.579784	0.000719	***
BMI	-0.21949	0.258009	-0.85071	0.398556	
ALT	0.12187	0.086079	1.415794	0.162374	
AST	-0.00302	0.038481	-0.07845	0.93775	
γ-GGT	-0.04195	0.057468	-0.72999	0.468437	
TBiL	-0.02094	0.113731	-0.18416	0.854556	
ALB	0.241975	0.239747	1.009291	0.317177	
Cr	-0.0159	0.084197	-0.1888	0.850932	
UA	-0.00921	0.017499	-0.52636	0.600719	
TG	1.83091	1.966854	0.930882	0.35591	
TC	-1.4933	2.210998	-0.6754	0.502203	
LDL-C	0.632385	2.425173	0.260759	0.795235	
FBG	2.055332	1.430231	1.437063	0.156264	

$^{***}P < 0.001$; *Cav-1* caveolin-1, *BMI* body mass index, *ALT* alanine aminotransferase, *AST* aspartate transaminase, *γ-GGT* γ-glutamine, *TBiL* total bilirubin, *ALB* albumin, *Cr* creatinine, *UA* uric acid, *TG* triglyceride, *TC* total cholesterol, *LDL-C* low-density lipoprotein-cholesterol, *FBG* fasting blood-glucose, *MLR* multiple linear regression

Table S6 Coefficients between Cav-1 and covariates (transferrin and confounders) in MLR model

Name	Estimate	Std. Error	*t* value	Pr (>|*t*|)	Significant
Intercept	-3.24495	6.779152	-0.47867	0.634039	
Transferrin	6.256441	1.658597	3.77213	0.000392	***
BMI	-0.19151	0.256014	-0.74805	0.457563	
ALT	0.119943	0.085226	1.407344	0.164852	
AST	-0.00453	0.038117	-0.1189	0.905781	
γ-GGT	-0.02612	0.056368	-0.4633	0.644946	
TBIL	-0.02889	0.112674	-0.25637	0.798606	
ALB	0.198604	0.239379	0.829662	0.410253	
Cr	-0.00941	0.083199	-0.11315	0.910315	
UA	-0.00827	0.017342	-0.47712	0.635134	
TG	1.578105	1.93638	0.814977	0.418538	
TC	-1.30442	2.185389	-0.59688	0.552992	
LDL-C	0.62516	2.399583	0.260529	0.795412	
FBG	1.968557	1.416346	1.389884	0.170065	

$^{***}P < 0.001$; *Cav-1* caveolin-1, *BMI* body mass index, *ALT* alanine aminotransferase, *AST* aspartate transaminase, *γ-GGT* γ-glutamine, *TBiL* total bilirubin, *ALB* albumin, *Cr* creatinine, *UA* uric acid, *TG* triglyceride, *TC* total cholesterol, *LDL-C* low-density lipoprotein-cholesterol, *FBG* fasting blood-glucose, *MLR* multiple linear regression

Table S7 Coefficients between Cav-1 and covariates (iron and confounders) in MLR model

**Name**	**Estimate**	**Std. Error**	***t* value**	**Pr (>|*t*|)**	**Significant**
Intercept	1.86292	8.071183	0.230811	0.818302624	
Iron	0.379384	0.190525	1.991259	0.051339187	
BMI	-0.35915	0.277398	-1.29471	0.2007332	
ALT	0.126266	0.09252	1.364746	0.177791782	
AST	0.00511	0.041125	0.124258	0.901556151	
γ-GGT	-0.00386	0.060923	-0.06332	0.949739343	
TBIL	-0.00629	0.121738	-0.05165	0.958991218	
ALB	0.228984	0.267737	0.855257	0.396053423	
Cr	-0.02076	0.091159	-0.22773	0.820683862	
UA	-0.01168	0.018791	-0.62159	0.536736478	
TG	1.131171	2.090802	0.541023	0.590637767	
TC	-0.89588	2.362784	-0.37916	0.706000988	
LDL-C	-0.79857	2.604093	-0.30666	0.760240128	
FBG	1.896286	1.537289	1.233526	0.222531874	

Cav-1 caveolin-1, *BMI* body mass index, *ALT* alanine aminotransferase, *AST* aspartate transaminase, *γ-GGT* γ-glutamine, *TBiL* total bilirubin, *ALB* albumin, *Cr* creatinine, *UA* uric acid, *TG* triglyceride, *TC* total cholesterol, *LDL-C* low-density lipoprotein-cholesterol, *FBG* fasting blood-glucose, *MLR* multiple linear regression

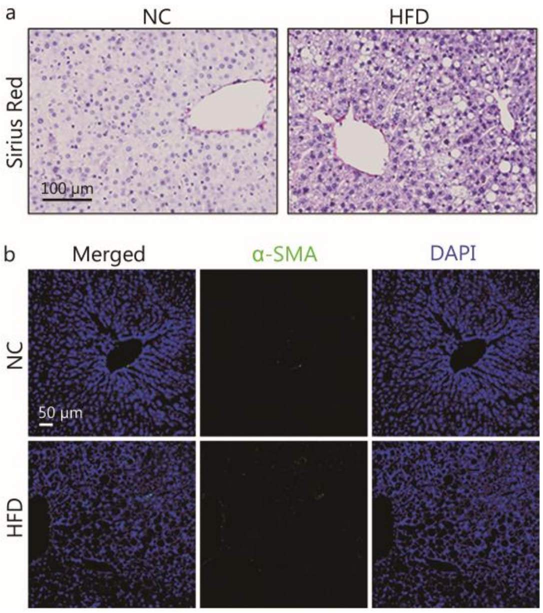

Fig. S1 Changes of liver fibrosis in mice after 12 weeks of high-fat diet (HFD). **a** Results of liver Sirius Red staining (*n* = 4). **b** Results of liver α-SMA immunofluorescence staining (*n* = 4). NC negative control, α-SMA alpha-smooth muscle actin

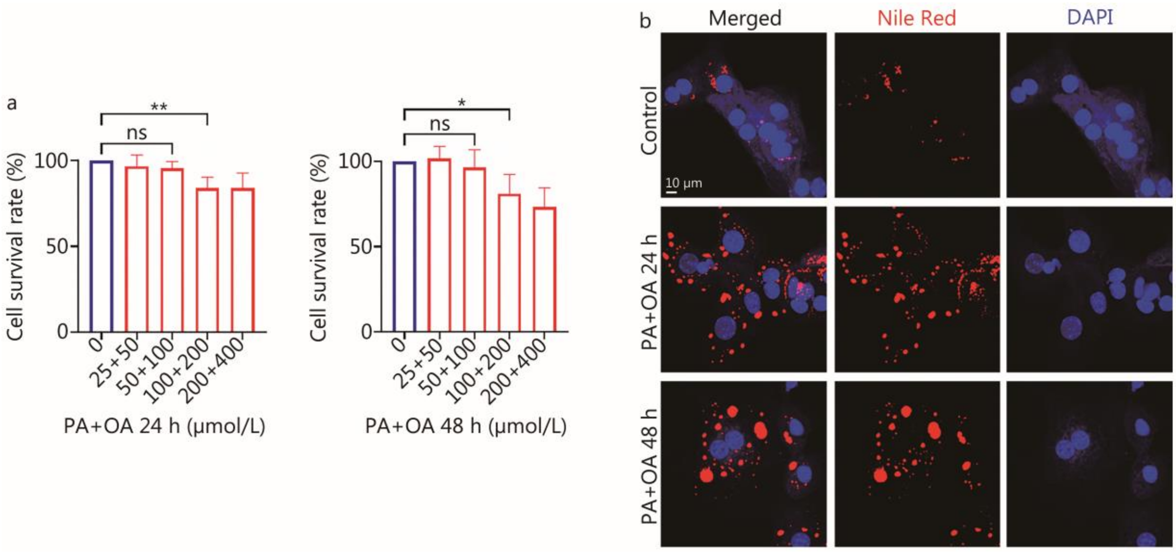

Fig. S2 PA + OA successfully constructed primary hepatocytes NAFLD model. **a** CCK-8 detection results of primary hepatocytes with or without PA + OA for 24 h and 48 h. **b** Nile Red staining results of primary hepatocytes with or without PA + OA. $^{*}P < 0.05$, $^{**}P < 0.01$, as determined by one-way ANOVA. All data were shown as the mean ± SD. ns non-significant, PA palmitic acid, OA oleic acid, NAFLD non-alcoholic fatty liver disease

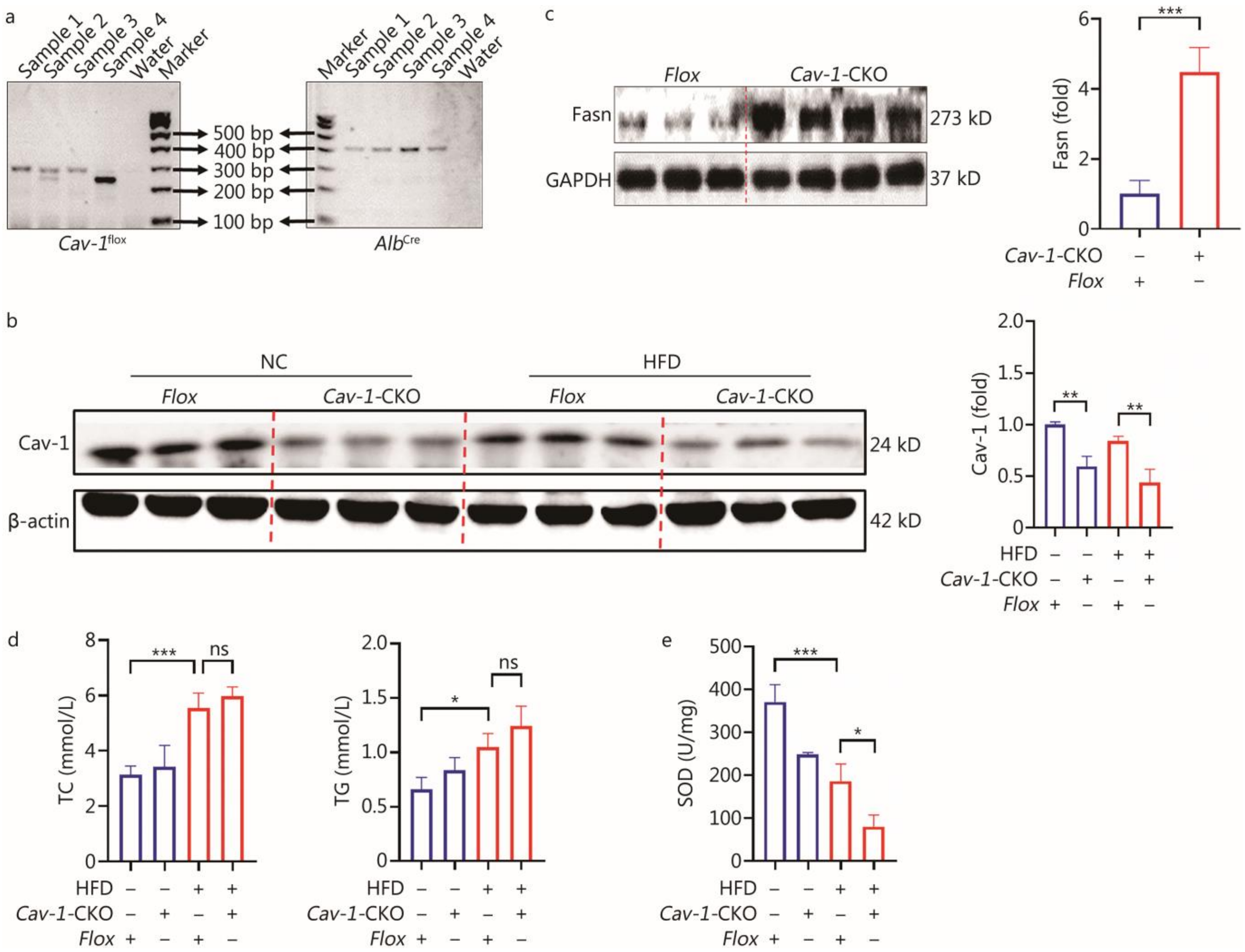

Fig. S3 Hepatocyte-specific *Cav-1* knockdown affected the development of NAFLD. **a** Cav-1^{flox} gene identification results (left panel; Cav-1^{flox}: 272 bp, WT: 232 bp), and Alb^{Cre} gene identification results (right panel; Alb^{Cre}: 390 bp, WT: 251 bp). **b** Western blotting results of Cav-1 in liver tissue between *Flox* mice and *Cav-1*-CKO mice with or without high-fat diet (HFD) (n = 3). **c** Western blotting detected the expression of Fasn in liver tissue between *Flox* mice and *Cav-1*-CKO mice ($n \geq 3$). **d** Concentration of serum TC and TG in different groups (n = 4). **e** Detection of SOD concentration in liver tissue (n = 3). $^{*}P < 0.05$, $^{**}P < 0.01$, $^{***}P< 0.001$, as determined by Student's t test analysis or one-way ANOVA. All data were shown as the mean ± SD. ns non-significant, Cav-1 caveolin-1, NAFLD non-alcoholic fatty liver disease, Fasn fatty acid synthase, TC total cholesterol, TG triglyceride, SOD superoxide dismutase

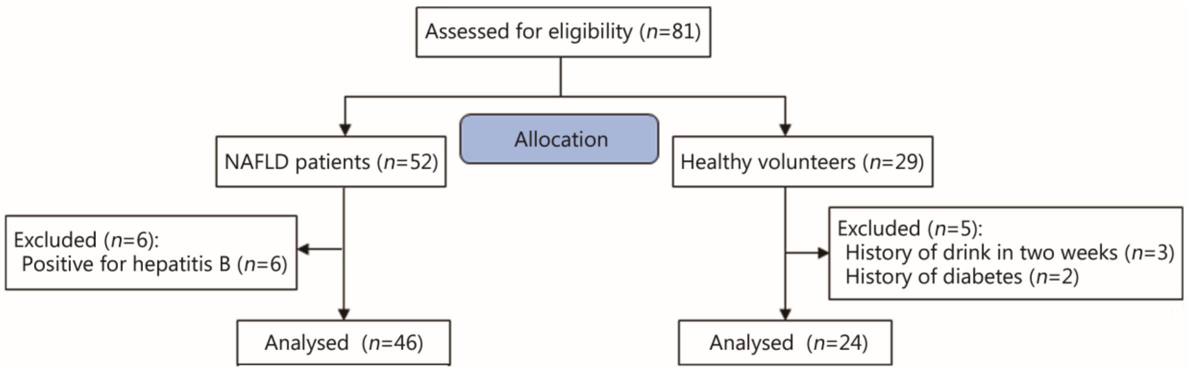

Fig. S4 Flow diagram of participants enrolled in the two study groups. Standard flow chart for enrollment and exclusion of healthy and NAFLD patient volunteers. NAFLD non-alcoholic fatty liver disease
